# Diagnostic Performance of Various Tests and Criteria Employed in Allergic Bronchopulmonary Aspergillosis: A Latent Class Analysis

**DOI:** 10.1371/journal.pone.0061105

**Published:** 2013-04-12

**Authors:** Ritesh Agarwal, Dipesh Maskey, Ashutosh Nath Aggarwal, Biman Saikia, Mandeep Garg, Dheeraj Gupta, Arunaloke Chakrabarti

**Affiliations:** 1 Department of Pulmonary Medicine, Postgraduate Institute of Medical Education and Research, Chandigarh, India; 2 Department of Immunopathology, Postgraduate Institute of Medical Education and Research, Chandigarh, India; 3 Department of Radiodiagnosis and Imaging, Postgraduate Institute of Medical Education and Research, Chandigarh, India; 4 Department of Medical Microbiology, Postgraduate Institute of Medical Education and Research, Chandigarh, India; Glaxo Smith Kline, Denmark

## Abstract

**Aim:**

The efficiency of various investigations and diagnostic criteria used in diagnosis of allergic bronchopulmonary aspergillosis (ABPA) remain unknown, primarily because of the lack of a gold standard. Latent class analysis (LCA) can provide estimates of sensitivity and specificity in absence of gold standard. Herein, we report the performance of various investigations and criteria employed in diagnosis of ABPA.

**Methods:**

Consecutive subjects with asthma underwent all the following investigations *Aspergillus* skin test, IgE levels (total and *A*.*fumigatus* specific), *Aspergillus* precipitins, eosinophil count, chest radiograph, and high-resolution computed tomography (HRCT) of the chest. We used LCA to estimate the performance of various diagnostic tests and criteria in identification of ABPA.

**Results:**

There were 372 asthmatics with a mean age of 35.9 years. The prevalence of Aspergillus sensitization was 53.2%. The sensitivity and specificity of various tests were *Aspergillus* skin test positivity (94.7%, 79.7%); IgE levels>1000 IU/mL (97.1%, 37.7%); *A*.*fumigatus* specific IgE levels>0.35 kUA/L (100%, 69.3%); *Aspergillus* precipitins (42.7%, 97.1%); eosinophil count>1000 cells/µL (29.5%, 93.1%); chest radiographic opacities (36.1%, 92.5%); bronchiectasis (91.9%, 80.9%); and, high-attenuation mucus (39.7%, 100%). The most accurate criteria was the Patterson criteria using six components followed by the Agarwal criteria. However, there was substantial decline in accuracy of the Patterson criteria if components of the criteria were either increased or decreased from six.

**Conclusions:**

*A*.*fumigatus* specific IgE levels and high-attenuation mucus were found to be the most sensitive and specific test respectively in diagnosis of ABPA. The Patterson criteria remain the best diagnostic criteria however they have good veridicality only if six criteria are used.

## Introduction

Allergic bronchopulmonary aspergillosis (ABPA) is a pulmonary disorder caused by immunologic reactions to antigens released by *Aspergillus fumigatus*, colonizing the airways of patients with asthma and cystic fibrosis. [1], [2] The disease presents with uncontrolled asthma, expectoration of mucus plugs, fleeting pulmonary opacities and bronchiectasis. [3] The community prevalence of ABPA complicating asthma is speculated to be about 1–2%, [4] with a global burden of about 5 million patients. [5] The prevalence of ABPA is even higher in the asthma clinics where it was estimated to be about 13%. [6] The diagnosis of ABPA is currently made on the combination of clinical, radiological and immunological findings. [7] Almost six decades have elapsed since its first description, yet there is no consensus on the diagnostic criteria for identification of ABPA. [8] The Patterson criteria are the most often used yardstick for diagnosis of ABPA, [9], [10] and utilize the following investigations in asthmatic patients, namely *Aspergillus* skin test, IgE levels (total and *A*. *fumigatus* specific), eosinophil count, *Aspergillus* precipitins, radiographic pulmonary opacities and bronchiectasis. However, there is no consensus on the number of parameters in the criteria required to make the diagnosis. Also, the criteria lay equal emphasis on all variables while some components of the criteria may be more important than others. To overcome some of the limitations of the Patterson criteria, we have proposed new criteria wherein greater emphasis has been laid on specific components in the diagnosis of ABPA, such as total and *A*. *fumigatus* specific IgE levels. [11] Unfortunately, the diagnostic performance of both the criteria remains unknown.

A major drawback in diagnosis of ABPA is the lack of gold standard for confirmation of diagnosis, which has made it virtually impossible to evaluate the sensitivity and specificity of the diagnostic criteria as a whole, and its individual components. As the currently used diagnostic criteria are a composite of the aforementioned diagnostic tests, [9], [11] conventional statistical methods would lead to erroneous estimates of sensitivity and specificity because investigations like skin test and IgE levels that are integral part of the criteria will have perfect (100%) sensitivity values. Latent class models using maximum likelihood (ML) methods can obtain direct estimates of sensitivity and specificity in the absence of an acceptable gold standard. [12], [13] Latent-class analysis (LCA) is based on the concept that the true disease status of the individual is unknown but certain probabilistic estimates can be made for ascertaining this position. Several studies have used LCA for the evaluation of diagnostic tests where no gold standard exists, and have found this model to provide realistic estimates of the performance of diagnostic tests. [14]–[16] In this study, we report the diagnostic accuracy of two diagnostic criteria (Patterson et al [9] and Agarwal et al [11]) and various tests (*Aspergillus* skin test, serum IgE levels [total and *A*. *fumigatus* specific], eosinophil count, *Aspergillus* precipitins, chest radiograph, bronchiectasis and high-attenuation mucus) used in identification of ABPA, using LCA.

## Materials and Methods

### Ethics Statement

The study was approved by the Institutes Ethics Committee, and a written informed consent was taken from all subjects. In case of minors/children participating in the study, written informed consent was obtained from the next of kin, caretakers, or guardians on behalf of the study participants.

### Patients

This was a prospective observational study conducted between July 2010 and July 2011 in the Chest Clinic of this Institute. Consecutive patients of bronchial asthma aged ≥15 years without a prior diagnosis of ABPA, were included in the study if they met two of the following three criteria: (a) history of recurrent attacks of chest tightness, breathlessness and cough; (b) documented wheeze on auscultation of the chest; and, (c) obstructive defect on spirometry with presence of bronchodilator reversibility. Patients without bronchodilator reversibility at the time of entry were included only if they had a documented bronchodilator reversibility at some point in their illness. Patients were excluded if they met any of the following criteria: (a) age>75 years; (b) prior diagnosis of ABPA or chronic obstructive pulmonary disease; (c) pregnancy; (d) other immunosuppressive states; (e) intake of systemic corticosteroids within last four weeks for controlling asthma; and, (f) failure to provide informed consent.

### Methods

All subjects underwent detailed clinical history and physical examination. The following details were recorded: age, gender, respiratory symptoms and history of atopy. The severity of asthma was categorized according the 2004 Global Initiative for Asthma (GINA) recommendations, which includes the effect of treatment on the disease severity. [17] Further, all subjects underwent the following investigations: intradermal test for *Aspergillus*, IgE levels (total), *A*. *fumigatus* specific IgE levels, serum precipitating antibodies against *A fumigatus*, chest radiograph and high-resolution computed tomography (HRCT) of the chest. All patients were screened with routine stool examination to exclude parasitic infestations.

#### *Aspergillus* skin test (AST)

Was performed by injecting *Aspergillus* antigen (0.2 mL of 100 PNU/mL; 1 PNU = 0.00001 mg/mL) intradermally in the forearm. Phosphate buffer saline (0.2 mL) served as the control. The injection site was examined every 15 minutes for one hour. An immediate cutaneous hyperreactivity was defined if a wheal and erythema developed within one minute, reached a maximum after 10 to 20 minutes, and resolved within one hour. The antigen arm skin reaction diameter had to be at least 8 mm greater than the control arm. [18].

#### Serum total IgE and *A. fumigatus*-specific IgE levels

The total IgE levels were assessed using quantitative enzyme-linked immunosorbent assay (Demeditec diagnostics GmbH, Kiel, Germany) while the *A*. *fumigatus* specific IgE levels were assayed using fluorescent enzyme immunoassay (UniCap Systems; Phadia, Stockholm, Sweden).

#### *Aspergillus* precipitins

Were detected using the Ouchterlony gel diffusion technique according to the method described by Longbottom and Pepys. [19].

#### Total eosinophil count

The total leucocyte count was initially determined using an auto-analyzer. The percentage of differential leucocyte count was ascertained by counting and classifying 100 WBCs on a peripheral blood smear. The total eosinophil count was obtained by multiplying the percentage with the total leucocyte count.

#### Pulmonary function test

Was performed on a dry rolling seal spirometer (Spiroflow; PK Morgan Ltd.; Kent, UK) to determine the lung function measurements and bronchodilator reversibility. Age, gender, height and spirometry data were recorded for all patients using computer software previously developed by us. [20].

#### HRCT of the chest

Was performed using a 16-row, multiple-detector CT scanner (LightSpeed Plus; GE Medical Systems; Slough, UK) with a matrix size of 512×512. The scans were obtained from the lung apex to the base using a scan time of three seconds while the patient was in the supine position at full end-tidal inspiration. Image acquisition was contiguous, and the images (1.25 mm at 10-mm intervals) were reconstructed using a high-spatial-frequency algorithm. The diagnosis of bronchiectasis on HRCT chest was made as per previously described criteria. [21].

#### Diagnosis of *Aspergillus* sensitization and ABPA

*Aspergillus* sensitization was defined by the presence of immediate cutaneous hyperreactivity on AST or the presence of *A*. *fumigatus* specific IgE>0.35 kUA/L. The diagnosis of ABPA (Table 1) was made according to the criteria proposed by Patterson et al. [9] and Agarwal et al. [11] As there is no consensus on the number of components of the Patterson criteria (Table 1) that is required to make a diagnosis of ABPA, ABPA was classified as present or absent according to the presence of five, six, seven or eight criteria for the purpose of this study. Patients with bronchiectasis who did not meet the criteria for ABPA underwent other investigations for ABPA according to the Chest Clinic protocol.

**Table 1:** Criteria for the diagnosis of allergic bronchopulmonary aspergillosis.

**Patterson criteria ** [**9**]
• Asthma, Immediate cutaneous hyperreactivity on *Aspergillus* skin test (type I reaction), Elevated serum IgE (>417 IU/mL), Elevated serum *A fumigatus* specific IgE (>0.35 kUA/L), Precipitating antibodies (IgG) in serum against *A*.*fumigatus*, Eosinophilia (>1000 cells/µL), Central bronchiectasis, Transient or fixed pulmonary opacities
• Diagnosis of ABPA was made on the presence of any five, six, seven or eight criteria
**Agarwal criteria ** [**11**]
• Obligatory criteria: Asthma, Elevated total IgE levels (>1000 IU/mL), Elevated IgE against *A*. *fumigatus* (>0.35 kUA/L)
• Other criteria: Type I *Aspergillus* skin test positive, Presence of serum precipitating antibodies against *A.fumigatus*, Radiographic pulmonary opacities (fixed/transient), Total eosinophil count>1000 cells/µL, Central bronchiectasis on high resolution computed tomography of the chest
• Diagnosis of ABPA was made on the presence of all obligatory criteria and three of five other criteria

#### Statistical analysis

was performed using the statistical package StatsDirect (version 2.7.8; StatsDirect Ltd, Cheshire, UK). Data are presented in a descriptive fashion as mean (95% confidence intervals [CI]) and number (percentage with 95% CI). The differences between continuous and categorical variables was analyzed using Mann-Whitney U and chi-square tests respectively. The agreement between two tests was determined using Cohen’s weighted kappa. [22] We used LCA (performed using the R-version of the TAGS software [23]) to estimate the accuracy of various diagnostic tests employed in the diagnosis of ABPA. The study population was divided into two groups namely uncontrolled and controlled asthma using the GINA criteria (categories of partly controlled and uncontrolled asthma merged to form the category of uncontrolled asthma for the purpose of this study). [24] Patients were cross-classified in both subsets of asthma based on the results of various investigations. A similar methodology was used to calculate the sensitivity and specificity of the two criteria used for diagnosis of ABPA. The Patterson criteria (five, six, seven or eight) were compared among themselves and with the Agarwal criteria (Table 1). The ML estimates for all tests were obtained using two iterative methods namely the Newton-Raphson algorithm and the expectation-maximization algorithm, which provided the sensitivity and specificity. [23] The CI obtained are the “percentile bootstrap” CI generated by bootstrapping the sample with 5000 samples independently with replacement from the data, within each subpopulation. The lower and upper 95% CI are then defined as the 2.5^th^ and 97.5^th^ percentiles of the bootstrap parameters. The fit of the LCA model (assumption of conditional independence) was performed by the goodness-of-fit test (likelihood ratio chi-square deviance test) followed by evaluation of residual correlations between tests. A good fit of the model is indicated by a p value>0.05 and the random distribution of the residuals near zero.

## Results

During the study period, 518 consecutive asthmatics were screened of which 388 patients met the criteria for inclusion in the study. Finally, 372 patients underwent all the investigations and formed the study group. There were 191 men and 181 women with a mean age of 35.9 years. The mean duration of asthma was 8.3 years. Majority had uncontrolled asthma (63.1%) with history of atopy being present in 23.4% of them. The baseline characteristics of the study population are shown in Table 2. The number of acute exacerbations requiring glucocorticoids and hospitalization, and the number of patients receiving moderate to high dose ICS were higher in uncontrolled asthma (Table 2). Similarly, the severity of asthma (corrected for treatment) as per the GINA criteria and the severity of obstruction on spirometry was also higher in patients with uncontrolled asthma.

**Table 2:** Baseline characteristics of the study population.

	Controlled asthma(n = 137)	Uncontrolled asthma (n = 235)	Total population (n = 372)	P value
Age, in years	34.7 (32.3–37.0)	36.5 (34.9–38.2)	35.9 (34.5–37.2)	.19
Male gender	68 (49.6%, 40.9–58.3)	111 (47.2%, 40.7–53.8)	179 (43.1%, 40.7–53.2)	.15
Duration of asthma, in years	8.2 (6.9–9.4)	8.4 (7.4–9.4)	8.3 (7.6–9.1)	.78
History of atopy	37 (27, 19.8–25.3)	50 (21.3, 16.2–27.1)	87 (23.4, 19.4–27.9)	.21
**Symptoms**				
Cough	132 (96.4%, 91.7–98.8)	219 (93.2%, 89.2–96.1)	351 (94.4%, 91.5–96.3)	.25
Breathlessness	124 (90.5%, 84.3–94.9)	215 (91.9%, 87.2–94.7)	339 (91.1%, 87.8–93.6)	.95
Wheeze	126 (91.9%, 86.1–95.9)	225 (95.7%, 92.3–97.9)	351 (94.4%, 91.5–96.3)	.13
Chest tightness	99 (72.3%, 63.9–79.6)	197 (83.8%, 78.5–88.3)	296 (79.6%, 75.2–83.4)	**.008**
Two or more exacerbations in last year requiring glucocorticoids	33 (24.1%, 17.2–32.1)	119 (50.6%, 44.1–57.2)	152 (40.9%, 35.9–45.9)	**.0001**
Hospitalization for asthma in last year	5 (3.6%, 1.2–8.3)	27 (11.5%, 7.8–16.3)	32 (8.6%, 6.2–11.9)	**.008**
**Treatment history**				
Intermittent SABA	19 (13.9%, 9.1–20.7)	25 (10.6%, 7.3–15.2)	44 (11.8%, 8.9–15.5)	**.001**
Low-dose ICS (<400 µg BDPE)	13 (9.5%, 5.6–15.6)	11 (4.7%, 2.6–8.2)	24 (6.5%, 4.4–9.4)	
Moderate dose ICS (400–800 µg BDPE) plus LABA	52 (37.9%, 30.3–46.3)	52 (22.1%, 17.3–27.9)	104 (23.6%–32.7)	
High-dose ICS (>800 µg BDPE) plus LABA	53 (38.7%, 30.9–47.1)	147 (62.6%, 56.2–68.5)	180 (48.4%, 43.4–53.5)	
**Severity of asthma**				
Mild intermittent	19 (13.9%, 8.5–20.8)	0	19 (5.1%, 3.3–7.8)	**.0001**
Mild persistent	14 (10.2%, 5.7–16.6)	18 (7.7%, 4.6–11.8)	32 (8.6%, 6.2–11.9)	
Moderate persistent	51 (37.2%, 29.1–45.9)	61 (25.9%, 20.5–32.1)	112 (30.1%, 25.7–34.9)	
Severe persistent	53 (38.7%, 30.5–47.4)	156 (66.4%, 59.9–72.4)	209 (56.2%, 51.1–61.1)	
**Lung function test**				
FEV_1_ (% predicted)	77.3 (73.1–81.6)	70.1 (66.9–73.4)	72.7 (70.2–75.3)	**.008**
FVC (% predicted)	85.6 (82.2–89.1)	80.6 (77.9–83.3)	82.5 (80.3–84.6)	**.02**
Bronchodilator reversibility	68 (49.6%, 40.9–58.3)	124 (52.8%, 46.2–59.3)	192 (51.6%, 46.5–56.7)	.52
**Severity of obstruction**				
Normal	69 (50.4%, 41.9–59.0)	83 (35.3%, 29.2–41.8)	152 (40.9%, 35.9–45.9)	**.015**
Mild	36 (26.2%, 19.1–34.5)	64 (27.2%, 21.6–33.4)	100 (26.9%, 22.6–31.6)	
Moderate	19 (13.9%, 8.6–20.8)	58 (24.7%, 19.3–30.7)	77 (20.7%, 16.9–25.1)	
Severe	13 (9.5%, 5.2–15.7)	30 (12.8%, 8.8–17.7)	43 (11.6%, 8.7–15.2)	
**Investigations for ABPA**				
Type 1 *Af* skin test positive	57 (41.6%, 33.3–50.3)	84 (35.7%, 29.6–42.2)	141 (37.9%, 33.1–42.9)	.26
IgE levels, IU/mL	4002 (3046–4959)	3608 (2914–4302)	3753 (3193–4314)	.51
*Af* specific IgE levels, kUA/L	9.72 (6.48–12.97)	9.74 (7.04–12.43)	9.73 (7.66–11.80)	.99
*Af* precipitins	19 (13.9%, 8.6–20.8)	28 (11.9%, 8.1–16.8)	47 (12.6%, 9.6–16.4)	.58
Total eosinophil count, cells/µL	701 (321–1080)	682 (529–835)	689 (520–858)	.92
Chest radiographic transient opacities	14 (10.2%, 5.7–16.6)	21 (8.9%, 5.6–13.3)	35 (9.4%, 6.8–12.8)	.68
HRCT evidence of bronchiectasis	47 (34.3%, 26.4–42.9)	90 (38.3%, 32.1–44.8)	137 (36.8%, 32.1–41.8)	.44
**Immunological subgroups**				
IgE levels>1000 ng/mL (417 IU/mL)	114 (83.2%, 75.9–89.1)	192 (81.7%, 76.2–86.4)	306 (82.3%, 78.1–85.8)	.71
IgE levels>1000 IU/mL	103 (75.2%, 67.1–82.2)	159 (67.7%, 61.3–73.6)	262 (70.4%, 65.6–74.9)	.13
*Af* specific IgE levels>0.35 kUA/L	69 (50.4%, 41.7–59.0)	107 (45.5%,39.0–52.1 )	176 (47.3%, 42.3–52.4 )	.37
Total eosinophil count>1000 cells/µL	18 (13.1%, 7.9–19.9)	35 (14.9%, 10.6–20.1)	53 (14.2%, 11.1–18.2)	.64

All values are represented as mean (95% CI) or Number (percentage, 95% CI).

Af- Aspergillus fumigatus; BDPE- beclomethasone dipropionate equivalent; FEV1- forced expiratory volume in the first second; FVC- forced vital capacity; ICS- inhaled corticosteroids; LABA- long acting β2 agonist; SABA- short acting β2 agonist;

### Evaluation of Tests for the Diagnosis of ABPA

The prevalence of *Aspergillus* sensitization was 53.2% (198/372), with the intradermal test being positive in 37.9% (141/372) and *A*. *fumigatus* specific IgE levels>0.35 kUA/L in 47.3% (176/372). There was moderate agreement between the *Aspergillus* skin test and specific IgE levels for detecting *Aspergillus* sensitization with the Cohen’s weighted κ being 56.9 (95% CI, 46.9–66.9). The frequency of asthmatics with IgE levels>1000 IU/mL and>417 IU/mL (>1000 ng/mL) was 70.4% and 82.3%, respectively. Bronchiectasis was diagnosed in 137 (36.8%) asthmatic patients (Table 2). The frequency of bronchiectasis was higher in patients with *Aspergillus* sensitization than those without (105/198 [53.0%] vs. 32/174 [18.4%], p = 0.0001). Of 137 patients with bronchiectasis, involvement of three or more lobes and six or more segments by bronchiectasis was seen in 88 and 70 patients, respectively. The cause of bronchiectasis was ABPA in 55 patients while in the remaining it was assumed secondary to asthma itself.

The observed frequencies of various tests for ABPA are shown in *Tables S1–3 of File S1* using three different models outlined in Table 3. The goodness-of-fit test for conditional independence was acceptable (p = 0.99 for all the models) and the residual correlations between the tests were randomly distributed around zero. The sensitivity and specificity of various investigations used in the diagnosis of ABPA using LCA is shown in Table 3. In all the models, *A*. *fumigatus* specific IgE levels>0.35 kUA/L had the best sensitivity value (100%). Although IgE levels had a sensitivity exceeding 92%, the specificity ranged from 23–39% implying a high false-positive rate. The investigation with best specificity was high-attenuation mucus (100%) however the sensitivity of this finding was only 40% (Table 3). The other tests with good specificity were *A*. *fumigatus* precipitins, total eosinophil count>1000 cells/µL and chest radiographic opacities; however the sensitivity was poor.

**Table 3:** Sensitivity and specificity of various diagnostic tests and diagnostic criteria used in the evaluation of allergic bronchopulmonary aspergillosis (ABPA) using latent class analysis.

	Sensitivity	Specificity
**Model 1**		
Type 1 *Aspergillus* skin test positive	88.0% (79.6–95.5)	85.9% (78.5–91.4)
IgE levels>1000 IU/mL	92.3% (86.1–97.7)	39.8% (33.3–46.6)
*A*.*fumigatus* specific IgE levels>0.35kUA/L	100% (100–100)	77.3% (68.5–85.4)
*A*.*fumigatus* precipitins	34.0% (24.0–46.6)	97.5% (95.4–99.2)
Total eosinophil count>1000 cells/µL	30.7% (21.8–41.1)	93.2% (89.8–96.2)
HRCT evidence of bronchiectasis	77.9% (66.2–93.5)	82.7% (66.2–93.5)
Chest radiographic transient opacities	23.7% (15.6–34.0)	96.9% (94.6–98.9)
**Model 2**		
Type 1 *Aspergillus* skin test positive	89.2% (80.7–96.7)	87.4% (80.4–92.2)
IgE levels>1000 ng/mL (417 IU/mL)	95.8% (91.2–99.4)	23.7% (18.3–30.0)
*A*.*fumigatus* specific IgE levels>0.35kUA/L	100% (100–100)	78.2% (69.6–85.3)
*A*.*fumigatus* precipitins	33.4% (23.9–47.3)	97.5% (95.2–99.2)
Total eosinophil count>1000 cells/µL	29.5% (21.0–40.0)	93.1% (89.6–96.3)
HRCT evidence of bronchiectasis	76.0% (65.7–91.3)	82.9% (77.9–87.9)
Chest radiographic transient opacities	22.4% (14.6–32.3)	96.9% (94.4–98.9)
**Model 3**		
Type 1 *Aspergillus* skin test positive	94.7% (87.7–100)	79.7% (72.6–88.6)
IgE levels>1000 IU/mL	97.1% (90.7–100)	37.7% (31.6–44.2)
*A*.*fumigatus* specific IgE levels>0.35kUA/L	100% (100–100)	69.3% (61.8–79.2)
*A*.*fumigatus* precipitins	42.7% (27.8–59.2)	97.1% (94.8–98.9)
Total eosinophil count>1000 cells/µL	36.1% (24.1–49.0)	92.5% (89.1–95.6)
HRCT evidence of bronchiectasis	91.9% (72.7–100)	80.9% (75.2–85.7)
Chest radiographic transient opacities	28.3% (16.9–41.7)	96.8% (94.5–98.8)
HRCT evidence of high-attenuationmucus	39.7% (23.9–58.4)	100% (100–100)

The values in parenthesis represent 2.5–97.5% bootstrap confidence intervals obtained by bootstrapping 5000 samples.

HRCT- high resolution computed tomography.

### Evaluation of Various Diagnostic Criteria for ABPA

ABPA was diagnosed in 56 and 55 patients respectively using six Patterson criteria and the criteria proposed by Agarwal et al with the observed frequencies of various diagnostic criteria shown in *Table S4 of File S1*. The goodness-of-fit test for conditional independence was acceptable (p = 0.99) and the residual correlations between the diagnostic criteria were randomly distributed around zero. Only seven patients met the diagnosis of ABPA by all the Patterson criteria. Once the Patterson criteria were decreased to five there was decline in the specificity while there was decline in the sensitivity if the criteria were increased beyond six (Table 4). Of these 56 patients, 55 had bronchiectasis and one was diagnosed as serological ABPA (ABPA-S) i.e. ABPA without bronchiectasis. Bronchiectasis was more extensive (median 11 segments; IQR, 6–14) and severe (varicose and cystic) in patients with ABPA while in patients with asthma (without ABPA) the bronchiectasis was less extensive (median 5 segments; IQR, 3–6) and cylindrical in all patients. All patients with ABPA were initiated on treatment with glucocorticoids and/or azoles. All went into remission with improvement in clinical and radiographic findings associated with decline in IgE levels by at least 35% at the end of three months.

**Table 4:** Accuracy of various diagnostic criteria for allergic bronchopulmonary aspergillosis (ABPA) using latent class analysis.

	No. ofpatients	Sensitivity	Specificity
Patterson criteria [9]			
At least 5 criteria	97	100% (100–100)	87% (83.2–90.6)
At least 6 criteria	56	100% (100–100)	100% (100–100)
At least 7 criteria	22	39.3% (26.4–52.1)	100% (100–100)
All 8 major criteria	07	12.5% (4.2–21.7)	100% (100–100)
Agarwal criteria [11]	55	96.4% (94.2–100)	100% (100–100)

The values in parenthesis represent 2.5–97.5% bootstrap confidence intervals obtained by bootstrapping 5000 samples.

## Discussion

The results of this study demonstrate the sensitivity and specificity of various tests and diagnostic criteria used in recognition of ABPA. To the best of our knowledge, this is the first study to provide estimates of performance of diagnostic tests and diagnostic criteria employed in detection of ABPA. The study found *A*. *fumigatus* specific IgE levels and high-attenuation mucus to be the most sensitive and specific tests respectively while varying sensitivity and specificity was observed for other tests. The diagnostic criteria of Patterson et al. proved to be the most accurate however at least six of the eight criteria need to be employed.

Cutaneous testing with *Aspergillus* antigen has been strongly advocated as a screening tool for ABPA with a negative result excluding ABPA. [18], [25]–[31] However, the results of our study suggest that *A*.*fumigatus* specific IgE levels would serve as the most sensitive test in screening asthmatic patients for ABPA. In fact, 6–12% of ABPA patients can be potentially missed if skin testing alone is utilized as the screening tool for ABPA. We found significant overlap of *A*. *fumigatus* specific IgE values between ABPA and asthma, and hence a poor specificity. Thus, *A*. *fumigatus* specific IgE level is a useful screening tool for ABPA in asthmatic patients. High-attenuation mucus is considered to be the most characteristic radiologic sign in ABPA,[28], [30], [32]–[34] and was found to be the most specific test in diagnosis of ABPA. The total IgE levels are elevated in many patients with asthma without ABPA, [35], [36] and in the current study elevated IgE levels had good sensitivity but poor specificity in diagnosis of ABPA as 70% of our population had IgE levels>1000 IU/mL. We further used different models with IgE cutoff values of 417 IU/mL and 1000 IU/mL as many groups use a total IgE>1000 IU/mL rather than >1000 ng/ml (417 IU/mL) in the diagnostic criteria.

While IgE levels were found to offer low specificity in diagnosis of ABPA, they remain a useful test during followup of patients with ABPA. [29] After treatment, there is clinicoradiological improvement along with decline in IgE levels by at least 25%. Similarly, increase in IgE levels by 50–100% of the baseline along with clinicoradiological worsening suggests an exacerbation of ABPA rather than exacerbation of asthma alone. [1] Bronchiectasis was observed in a large number of asthmatic patients with higher prevalence in patients with *Aspergillus* sensitization. These results are in accordance with previous studies wherein the reported prevalence of bronchiectasis in asthmatic patients range from 18–52%; [37]–[42] the prevalence being higher in patients with severe compared to mild asthma. [43] Although bronchiectasis affecting three or more lobes, centrilobular nodules, and mucoid impaction on CT chest suggests ABPA, [39] a recent study noted all these findings in *Aspergillus*-sensitization without ABPA. [42].

No single test had both good sensitivity and specificity, hence composite of other tests should be utilized for confirmation of ABPA. The criteria proposed by Agarwal et al had good sensitivity and specificity, however the criteria still missed a single patient who met all other criteria for ABPA but the IgE value was <1000 IU/mL. An occasional patient with ABPA could manifest with IgE values <1000 IU/mL and this point should be kept in mind while investigating asthmatic patients for ABPA. [44] As there is no consensus on the number of Patterson criteria that should be used for diagnosis, several studies have used even five criteria. [27], [28], [45], [46] This study suggests that there is decline in specificity if five criteria are employed and fall in sensitivity if seven criteria are employed. In fact, in this study only seven patients with ABPA met all the eight major Patterson criteria. Thus, six or higher Patterson criteria offer the best diagnostic performance in identification of ABPA.

The differentiation between ABPA and *Aspergillus* sensitized asthma is important because patients with ABPA generally require treatment with corticosteroids to control the immune activity while those with *Aspergillus* sensitization do not. [47] This makes it imperative to know the diagnostic performance of individual tests and various diagnostic criteria. In fact, one study estimated that one-third of medical articles dealing with evaluation of diagnostic tests use no gold standard, and thus it is not known whether their use improves clinical outcomes. [48], [49] Imperfect estimates of test accuracy can obviously have serious clinical consequences with a false-positive test leading to overtreatment (and risk of unnecessary adverse effects of treatment) while a false-negative result can cause adverse consequences of the disease. We used LCA, a statistical modeling technique which examines associations between observed variables (in our case the different diagnostic tests and diagnostic criteria) that imperfectly measure a non-observable or latent variable. [13] In the current study, the true diagnosis of ABPA was considered as the latent variable in two categories namely ‘controlled’ and ‘uncontrolled’ asthma. The ML model that we used in our study is restricted to binary test results (one value signifying positive test and the other a negative test). The biggest advantage of LCA is obviously the evaluation of diagnostic accuracy of test(s) in the absence of gold standard, which made it possible to estimate the precision of diagnostic tests and diagnostic criteria used in recognition of ABPA.

Finally, our study is not without limitations. The major limitation is the small sample size and the conduct of the study at a single tertiary care referral center although a minimum of three tests and roughly 100 observations are required for a model of conditional independence. [50] Thus, studies with similar design need to be conducted at different centers with a larger sample size. Another limitation is that we used an in-house *Aspergillus* antigen for diagnosis of AH although this has been standardized against aspergillin (commercial antigen). In fact, after analyzing both cases and controls, an 8 mm cutoff has been chosen to declare skin-test positivity. The test has no false-positivity, as in one study the skin test was negative in all the 100 healthy controls tested. [11] The strength of our study is the robust study design and the fact that the sampled population included an appropriate spectrum of the disease.

In conclusion, the results of this study suggest that *A*. *fumigatus* specific IgE levels are the most sensitive test in diagnosis of ABPA, and can be used as a screening test for ABPA. On the other hand, high-attenuation mucus was found to be the most specific test. As no single test had both good sensitivity and specificity, series of tests need to be performed for confirmation of ABPA. The diagnostic criteria proposed by Patterson et al. remain the gold standard for diagnosis of ABPA if six criteria are used. However, there is a decline in diagnostic accuracy of the Patterson criteria if the number of criterion is either increased or decreased.

## Supporting Information

File S1 **Tables S1–S4.**
(DOC)

## References

[1] Agarwal R (2009) Allergic bronchopulmonary aspergillosis. Chest 135: 805–826. 19265090 10.1378/chest.08-2586

[2] Tillie-Leblond I, Tonnel AB (2005) Allergic bronchopulmonary aspergillosis. Allergy 60: 1004–1013. 15969680 10.1111/j.1398-9995.2005.00887.x

[3] Agarwal R, Chakrabarti A (2010) Clinical manifestations and natural history of allergic bronchopulmonary aspergillosis. In: Pasqualotto AC, editor. Aspergillosis: From Diagnosis to Prevention. New York: Springer. 707–724.

[4] Agarwal R, Chakrabarti A (2010) Epidemiology of allergic bronchopulmonary aspergillosis. In: Pasqualotto AC, editor. Aspergillosis: From Diagnosis to Prevention. New York: Springer. 671–688.

[5] Denning DW, Pleuvry A, Cole DC (2013) Global burden of allergic bronchopulmonary aspergillosis with asthma and its complication chronic pulmonary aspergillosis in adults. Med Mycol In Press doi: 10.3109/13693786.13692012.13738312. 10.3109/13693786.2012.738312 23210682

[6] Agarwal R, Aggarwal AN, Gupta D, Jindal SK (2009) *Aspergillus* hypersensitivity and allergic bronchopulmonary aspergillosis in patients with bronchial asthma: systematic review and meta-analysis. Int J Tuberc Lung Dis 13: 936–944. 19723372

[7] Hogan C, Denning DW (2011) Allergic bronchopulmonary aspergillosis and related allergic syndromes. Semin Respir Crit Care Med 32: 682–692. 22167396 10.1055/s-0031-1295716

[8] Agarwal R (2010) Controversies in allergic bronchopulmonary aspergillosis. Int J Respir Care 6: 53–54, 56–63.

[9] Rosenberg M, Patterson R, Mintzer R, Cooper BJ, Roberts M, et al (1977) Clinical and immunologic criteria for the diagnosis of allergic bronchopulmonary aspergillosis. Ann Intern Med 86: 405–414. 848802 10.7326/0003-4819-86-4-405

[10] Patterson R, Greenberger PA, Halwig JM, Liotta JL, Roberts M (1986) Allergic bronchopulmonary aspergillosis. Natural history and classification of early disease by serologic and roentgenographic studies. Arch Intern Med 146: 916–918. 3516103 10.1001/archinte.146.5.916

[11] Agarwal R, Hazarika B, Gupta D, Aggarwal AN, Chakrabarti A, et al (2010) Aspergillus hypersensitivity in patients with chronic obstructive pulmonary disease: COPD as a risk factor for ABPA? Med Mycol 48: 988–994. 20370368 10.3109/13693781003743148

[12] Hui SL, Walter SD (1980) Estimating the error rates of diagnostic tests. Biometrics 36: 167–171. 7370371

[13] Rindskopf D, Rindskopf W (1986) The value of latent class analysis in medical diagnosis. Stat Med 5: 21–27. 3961312 10.1002/sim.4780050105

[14] Nascimento MC, de Souza VA, Sumita LM, Freire W, Munoz F, et al (2007) Comparative study of Kaposi’s sarcoma-associated herpesvirus serological assays using clinically and serologically defined reference standards and latent class analysis. J Clin Microbiol 45: 715–720. 17182752 10.1128/JCM.01264-06 PMC1829101

[15] Girardi E, Angeletti C, Puro V, Sorrentino R, Magnavita N, et al.. (2009) Estimating diagnostic accuracy of tests for latent tuberculosis infection without a gold standard among healthcare workers. Euro Surveillance 14: pii = 19373. 19883555

[16] Machado de Assis TS, Rabello A, Werneck GL (2012) Latent class analysis of diagnostic tests for visceral leishmaniasis in Brazil. Trop Med Int Health In Press: doi:10.1111/j.1365–3156.2012.03064.x. 10.1111/j.1365-3156.2012.03064.x 22897740

[17] Global Initiative for Asthma (GINA)- Global strategy for asthma management and prevention (2004). Available: http://wwwginasthmaorg/documents/5/documents_variants/27 Accessed 2013 March 12.

[18] Agarwal R, Noel V, Aggarwal AN, Gupta D, Chakrabarti A (2011) Clinical significance of Aspergillus sensitisation in bronchial asthma. Mycoses 54: e531–538. 21605181 10.1111/j.1439-0507.2010.01971.x

[19] Longbottom JL, Pepys J (1964) Pulmonary aspergillosis: Diagnostic and immunological significance of antigens and C-Substance in Aspergillus Fumigatus. J Pathol Bacteriol 88: 141–151. 14194971 10.1002/path.1700880119

[20] Aggarwal AN, Gupta D, Jindal SK (2002) Development of a simple computer program for spirometry interpretation. J Assoc Physicians India 50: 567–570. 12164412

[21] Reiff DB, Wells AU, Carr DH, Cole PJ, Hansell DM (1995) CT findings in bronchiectasis: limited value in distinguishing between idiopathic and specific types. AJR Am J Roentgenol 165: 261–267. 7618537 10.2214/ajr.165.2.7618537

[22] Sim J, Wright CC (2005) The kappa statistic in reliability studies: use, interpretation, and sample size requirements. Phys Ther 85: 257–268. 15733050

[23] Pouillot R, Gerbier G, Gardner IA (2002) “TAGS”. a program for the evaluation of test accuracy in the absence of a gold standard. Prev Vet Med 53: 67–81. 11821138 10.1016/s0167-5877(01)00272-0

[24] Global Initiative for Asthma (GINA). Global strategy for asthma management and prevention (2012). Available: http://www.ginasthma.org/local/uploads/files/GINA_Report_2012Feb13.pdf.Accessed 2013 March 12.

[25] Greenberger PA, Patterson R (1988) Allergic bronchopulmonary aspergillosis and the evaluation of the patient with asthma. J Allergy Clin Immunol 81: 646–650. 3356845 10.1016/0091-6749(88)91034-2

[26] Eaton T, Garrett J, Milne D, Frankel A, Wells AU (2000) Allergic bronchopulmonary aspergillosis in the asthma clinic. A prospective evaluation of CT in the diagnostic algorithm. Chest 118: 66–72. 10893361 10.1378/chest.118.1.66

[27] Agarwal R, Gupta D, Aggarwal AN, Behera D, Jindal SK (2006) Allergic bronchopulmonary aspergillosis: lessons from 126 patients attending a chest clinic in north India. Chest 130: 442–448. 16899843 10.1378/chest.130.2.442

[28] Agarwal R, Gupta D, Aggarwal AN, Saxena AK, Chakrabarti A, et al (2007) Clinical significance of hyperattenuating mucoid impaction in allergic bronchopulmonary aspergillosis: an analysis of 155 patients. Chest 132: 1183–1190. 17646221 10.1378/chest.07-0808

[29] Agarwal R, Gupta D, Aggarwal AN, Saxena AK, Saikia B, et al (2010) Clinical significance of decline in serum IgE Levels in allergic bronchopulmonary aspergillosis. Respir Med 104: 204–210. 19800210 10.1016/j.rmed.2009.09.005

[30] Agarwal R, Khan A, Gupta D, Aggarwal AN, Saxena AK, et al (2010) An alternate method of classifying allergic bronchopulmonary aspergillosis based on high-attenuation mucus. PLoS One 5: e15346. 21179536 10.1371/journal.pone.0015346 PMC3002283

[31] Agarwal R, Nath A, Aggarwal AN, Gupta D, Chakrabarti A (2010) Aspergillus hypersensitivity and allergic bronchopulmonary aspergillosis in patients with acute severe asthma in a respiratory intensive care unit in North India. Mycoses 53: 138–143. 19207831 10.1111/j.1439-0507.2008.01680.x

[32] Agarwal R, Aggarwal AN, Gupta D (2006) High-attenuation mucus in allergic bronchopulmonary aspergillosis: another cause of diffuse high-attenuation pulmonary abnormality. AJR Am J Roentgenol 186: 904. 16498131 10.2214/AJR.05.0125

[33] Agarwal R (2010) High attenuation mucoid impaction in allergic bronchopulmonary aspergillosis. World J Radiol 2: 41–43. 21160739 10.4329/wjr.v2.i1.41 PMC2999313

[34] Agarwal R, Khan A, Garg M, Aggarwal AN, Gupta D (2011) Pictorial essay: Allergic bronchopulmonary aspergillosis. Indian J Radiol Imaging 21: 242–252. 22223932 10.4103/0971-3026.90680 PMC3249935

[35] Gleich GJ, Averbeck AK, Swedlund HA (1971) Measurement of IgE in normal and allergic serum by radioimmunoassay. J Lab Clin Med 77: 690–698. 4101838

[36] Malo JL, Longbottom J, Mitchell J, Hawkins R, Pepys J (1977) Studies in chronic allergic bronchopulmonary aspergillosis. 3. Immunological findings. Thorax 32: 269–274. 329461 10.1136/thx.32.3.269 PMC470596

[37] Angus RM, Davies ML, Cowan MD, McSharry C, Thomson NC (1994) Computed tomographic scanning of the lung in patients with allergic bronchopulmonary aspergillosis and in asthmatic patients with a positive skin test to Aspergillus fumigatus. Thorax 49: 586–589. 8016796 10.1136/thx.49.6.586 PMC474951

[38] Grenier P, Mourey-Gerosa I, Benali K, Brauner MW, Leung AN, et al (1996) Abnormalities of the airways and lung parenchyma in asthmatics: CT observations in 50 patients and inter- and intraobserver variability. European Radiology 6: 199–206. 8797980 10.1007/BF00181147

[39] Ward S, Heyneman L, Lee MJ, Leung AN, Hansell DM, et al (1999) Accuracy of CT in the diagnosis of allergic bronchopulmonary aspergillosis in asthmatic patients. AJR Am J Roentgenol 173: 937–942. 10511153 10.2214/ajr.173.4.10511153

[40] Jensen SP, Lynch DA, Brown KK, Wenzel SE, Newell JD (2002) High-resolution CT features of severe asthma and bronchiolitis obliterans. Clin Radiol 57: 1078–1085. 12475532 10.1053/crad.2002.1104

[41] Fairs A, Agbetile J, Hargadon B, Bourne M, Monteiro WR, et al (2010) IgE sensitisation to Aspergillus fumigatus is sssociated with reduced lung function in asthma. Am J Respir Crit Care Med 182: 1362–1368. 20639442 10.1164/rccm.201001-0087OC PMC3029929

[42] Menzies D, Holmes L, McCumesky G, Prys-Picard C, Niven R (2011) Aspergillus sensitization is associated with airflow limitation and bronchiectasis in severe asthma. Allergy 66: 679–685. 21261660 10.1111/j.1398-9995.2010.02542.x

[43] Harmanci E, Kebapci M, Metintas M, Ozkan R (2002) High-resolution computed tomography findings are correlated with disease severity in asthma. Respiration 69: 420–426. 12232449 10.1159/000064018

[44] Hamburger RN (2005) Allergic Bronchopulmonary Aspergillosis with Normal Serum IgE in a Child with Cystic Fibrosis. Pediatr Asthma, Allerg Immunol 18: 46–47.

[45] Kumar R, Gaur SN (2000) Prevalence of allergic bronchopulmonary aspergillosis in patients with bronchial asthma. Asian Pac J Allergy Immunol 18: 181–185. 11316037

[46] Ghosh T, Dey A, Biswas D, Chatterjee S, Haldar N, et al (2010) Aspergillus hypersensitivity and allergic bronchopulmonary aspergillosis among asthma patients in eastern India. J Indian Med Assoc 108: 863–865. 21661466

[47] Agarwal R (2011) Severe asthma with fungal sensitization. Curr Allergy Asthma Rep 11: 403–413. 21789577 10.1007/s11882-011-0217-4

[48] Sheps SB, Schechter MT (1984) The assessment of diagnostic tests. A survey of current medical research. JAMA 252: 2418–2422. 6481928

[49] Guyatt GH, Tugwell PX, Feeny DH, Haynes RB, Drummond M (1986) A framework for clinical evaluation of diagnostic technologies. CMAJ 134: 587–594. 3512062 PMC1490902

[50] Boelaert M, el Safi S, Goetghebeur E, Gomes-Pereira S, Le Ray D, et al (1999) Latent class analysis permits unbiased estimates of the validity of DAT for the diagnosis of visceral leishmaniasis. Trop Med Int Health 4: 395–401. 10402977 10.1046/j.1365-3156.1999.00421.x

