# Supplementary material for: Diagnostic Performance of Various Tests and Criteria Employed in Allergic Bronchopulmonary Aspergillosis: A Latent Class Analysis
Source: PLoS One. 2013 Apr 12;8(4):e61105. doi: 10.1371/journal.pone.0061105 (PMC3625190; doi:10.1371/journal.pone.0061105)
Supplement: File S1 — Tables S1–S4. (DOC) [file pone.0061105.s001.doc]

**SUPPLEMENTARY DATA**

**Table S1**: Observed frequencies of allergic bronchopulmonary aspergillosis (ABPA) test patterns estimated by latent class analysis (model 1 with IgE values >1000 IU/mL) [Af- *Aspergillus fumigatus*]

| Aspergillus skin test | IgE >1000 IU/mL | Af IgE >0.35 kUA/L | Af precipitins | Total eosinophil count | Central bronchiectasis | Chest radiographic transient opacities | Observed frequency |
| --- | --- | --- | --- | --- | --- | --- | --- |
| Controlled asthma |  |  |  |  |  |  |  |
| 0 | 0 | 0 | 0 | 0 | 0 | 0 | 17 |
| 1 | 0 | 0 | 0 | 0 | 0 | 0 | 2 |
| 0 | 1 | 0 | 0 | 0 | 0 | 0 | 25 |
| 1 | 1 | 0 | 0 | 0 | 0 | 0 | 4 |
| 0 | 0 | 1 | 0 | 0 | 0 | 0 | 1 |
| 1 | 0 | 1 | 0 | 0 | 0 | 0 | 2 |
| 0 | 1 | 1 | 0 | 0 | 0 | 0 | 15 |
| 1 | 1 | 1 | 0 | 0 | 0 | 0 | 13 |
| 0 | 0 | 0 | 1 | 0 | 0 | 0 | 3 |
| 1 | 0 | 1 | 1 | 0 | 0 | 0 | 1 |
| 0 | 0 | 0 | 0 | 1 | 0 | 0 | 2 |
| 0 | 1 | 0 | 0 | 1 | 0 | 0 | 2 |
| 0 | 1 | 1 | 0 | 1 | 0 | 0 | 1 |
| 1 | 1 | 1 | 0 | 1 | 0 | 0 | 1 |
| 0 | 0 | 0 | 0 | 0 | 1 | 0 | 2 |
| 1 | 0 | 0 | 0 | 0 | 1 | 0 | 1 |
| 0 | 1 | 0 | 0 | 0 | 1 | 0 | 4 |
| 1 | 1 | 0 | 0 | 0 | 1 | 0 | 1 |
| 0 | 0 | 1 | 0 | 0 | 1 | 0 | 0 |
| 1 | 0 | 1 | 0 | 0 | 1 | 0 | 1 |
| 0 | 1 | 1 | 0 | 0 | 1 | 0 | 1 |
| 1 | 1 | 1 | 0 | 0 | 1 | 0 | 10 |
| 1 | 1 | 1 | 1 | 0 | 1 | 0 | 6 |
| 0 | 1 | 0 | 0 | 1 | 1 | 0 | 3 |
| 1 | 1 | 1 | 0 | 1 | 1 | 0 | 4 |
| 1 | 1 | 1 | 1 | 1 | 1 | 0 | 1 |
| 1 | 0 | 0 | 0 | 0 | 0 | 1 | 1 |
| 0 | 1 | 1 | 0 | 0 | 0 | 1 | 1 |
| 0 | 0 | 0 | 0 | 0 | 1 | 1 | 1 |
| 1 | 1 | 1 | 0 | 0 | 1 | 1 | 2 |
| 0 | 1 | 1 | 1 | 0 | 1 | 1 | 1 |
| 1 | 1 | 1 | 1 | 0 | 1 | 1 | 4 |
| 0 | 1 | 1 | 0 | 1 | 1 | 1 | 1 |
| 1 | 1 | 1 | 1 | 1 | 1 | 1 | 3 |
| Uncontrolled asthma |  |  |  |  |  |  |  |
| 0 | 0 | 0 | 0 | 0 | 0 | 0 | 40 |
| 1 | 0 | 0 | 0 | 0 | 0 | 0 | 9 |
| 0 | 1 | 0 | 0 | 0 | 0 | 0 | 45 |
| 1 | 1 | 0 | 0 | 0 | 0 | 0 | 2 |
| 0 | 0 | 1 | 0 | 0 | 0 | 0 | 3 |
| 1 | 0 | 1 | 0 | 0 | 0 | 0 | 4 |
| 0 | 1 | 1 | 0 | 0 | 0 | 0 | 16 |
| 1 | 1 | 1 | 0 | 0 | 0 | 0 | 8 |
| 0 | 0 | 0 | 1 | 0 | 0 | 0 | 1 |
| 0 | 1 | 0 | 1 | 0 | 0 | 0 | 1 |
| 1 | 1 | 1 | 1 | 0 | 0 | 0 | 1 |
| 0 | 0 | 0 | 0 | 1 | 0 | 0 | 1 |
| 0 | 1 | 0 | 0 | 1 | 0 | 0 | 2 |
| 1 | 0 | 1 | 0 | 1 | 0 | 0 | 1 |
| 0 | 1 | 1 | 0 | 1 | 0 | 0 | 2 |
| 1 | 1 | 1 | 0 | 1 | 0 | 0 | 3 |
| 1 | 1 | 1 | 1 | 1 | 0 | 0 | 1 |
| 0 | 0 | 0 | 0 | 0 | 1 | 0 | 4 |
| 0 | 1 | 0 | 0 | 0 | 1 | 0 | 14 |
| 1 | 1 | 0 | 0 | 0 | 1 | 0 | 1 |
| 0 | 0 | 1 | 0 | 0 | 1 | 0 | 1 |
| 1 | 0 | 1 | 0 | 0 | 1 | 0 | 2 |
| 0 | 1 | 1 | 0 | 0 | 1 | 0 | 8 |
| 1 | 1 | 1 | 0 | 0 | 1 | 0 | 17 |
| 0 | 0 | 0 | 1 | 0 | 1 | 0 | 1 |
| 1 | 0 | 1 | 1 | 0 | 1 | 0 | 1 |
| 0 | 1 | 1 | 1 | 0 | 1 | 0 | 1 |
| 1 | 1 | 1 | 1 | 0 | 1 | 0 | 6 |
| 0 | 0 | 0 | 0 | 1 | 1 | 0 | 2 |
| 1 | 0 | 0 | 0 | 1 | 1 | 0 | 1 |
| 1 | 0 | 1 | 0 | 1 | 1 | 0 | 1 |
| 0 | 1 | 1 | 0 | 1 | 1 | 0 | 1 |
| 1 | 1 | 1 | 0 | 1 | 1 | 0 | 6 |
| 1 | 0 | 1 | 1 | 1 | 1 | 0 | 1 |
| 1 | 1 | 1 | 1 | 1 | 1 | 0 | 6 |
| 0 | 0 | 0 | 0 | 0 | 0 | 1 | 1 |
| 0 | 1 | 0 | 0 | 0 | 0 | 1 | 1 |
| 0 | 1 | 1 | 0 | 0 | 0 | 1 | 1 |
| 1 | 1 | 1 | 0 | 0 | 0 | 1 | 1 |
| 0 | 1 | 0 | 0 | 1 | 0 | 1 | 1 |
| 0 | 0 | 0 | 0 | 0 | 1 | 1 | 1 |
| 0 | 1 | 1 | 0 | 0 | 1 | 1 | 1 |
| 1 | 1 | 1 | 0 | 0 | 1 | 1 | 4 |
| 0 | 0 | 1 | 1 | 0 | 1 | 1 | 1 |
| 0 | 1 | 1 | 1 | 0 | 1 | 1 | 1 |
| 1 | 1 | 1 | 1 | 0 | 1 | 1 | 2 |
| 0 | 1 | 1 | 0 | 1 | 1 | 1 | 1 |
| 1 | 1 | 1 | 0 | 1 | 1 | 1 | 2 |
| 1 | 1 | 1 | 1 | 1 | 1 | 1 | 4 |

**Table S2**: Observed frequencies of allergic bronchopulmonary aspergillosis (ABPA) test patterns estimated by latent class analysis (model 1 with IgE values >417 IU/mL) [Af- Aspergillus fumigatus]

| Aspergillus skin test | IgE >1000 IU/mL | Af IgE >0.35 kUA/L | Af precipitins | Total eosinophil  count | Central bronchiectasis | Chest radiographic transient opacities | Observed frequency |
| --- | --- | --- | --- | --- | --- | --- | --- |
| Controlled asthma |  |  |  |  |  |  |  |
| 0 | 0 | 0 | 0 | 0 | 0 | 0 | 13 |
| 1 | 0 | 0 | 0 | 0 | 0 | 0 | 1 |
| 0 | 1 | 0 | 0 | 0 | 0 | 0 | 29 |
| 1 | 1 | 0 | 0 | 0 | 0 | 0 | 5 |
| 0 | 1 | 1 | 0 | 0 | 0 | 0 | 15 |
| 1 | 1 | 1 | 0 | 0 | 0 | 0 | 15 |
| 0 | 0 | 0 | 1 | 0 | 0 | 0 | 2 |
| 0 | 1 | 0 | 1 | 0 | 0 | 0 | 1 |
| 1 | 0 | 1 | 1 | 0 | 0 | 0 | 1 |
| 0 | 0 | 0 | 0 | 1 | 0 | 0 | 2 |
| 0 | 1 | 0 | 0 | 1 | 0 | 0 | 2 |
| 0 | 1 | 1 | 0 | 1 | 0 | 0 | 1 |
| 1 | 1 | 1 | 0 | 1 | 0 | 0 | 1 |
| 0 | 0 | 0 | 0 | 0 | 1 | 0 | 1 |
| 1 | 0 | 0 | 0 | 0 | 1 | 0 | 1 |
| 0 | 1 | 0 | 0 | 0 | 1 | 0 | 5 |
| 1 | 1 | 0 | 0 | 0 | 1 | 0 | 1 |
| 0 | 1 | 1 | 0 | 0 | 1 | 0 | 2 |
| 1 | 1 | 1 | 0 | 0 | 1 | 0 | 11 |
| 1 | 1 | 1 | 1 | 0 | 1 | 0 | 6 |
| 0 | 1 | 0 | 0 | 1 | 1 | 0 | 3 |
| 1 | 1 | 1 | 0 | 1 | 1 | 0 | 4 |
| 1 | 1 | 1 | 1 | 1 | 1 | 0 | 1 |
| 1 | 0 | 0 | 0 | 0 | 0 | 1 | 1 |
| 0 | 1 | 1 | 0 | 0 | 0 | 1 | 1 |
| 1 | 1 | 1 | 0 | 0 | 1 | 1 | 2 |
| 0 | 1 | 1 | 1 | 0 | 1 | 1 | 1 |
| 1 | 1 | 1 | 1 | 0 | 1 | 1 | 4 |
| 0 | 1 | 1 | 0 | 1 | 1 | 1 | 1 |
| 1 | 1 | 1 | 1 | 1 | 1 | 1 | 3 |
| Uncontrolled asthma |  |  |  |  |  |  |  |
| 0 | 0 | 0 | 0 | 0 | 0 | 0 | 25 |
| 1 | 0 | 0 | 0 | 0 | 0 | 0 | 5 |
| 0 | 1 | 0 | 0 | 0 | 0 | 0 | 60 |
| 1 | 1 | 0 | 0 | 0 | 0 | 0 | 5 |
| 0 | 0 | 1 | 0 | 0 | 0 | 0 | 1 |
| 1 | 0 | 1 | 0 | 0 | 0 | 0 | 2 |
| 0 | 1 | 1 | 0 | 0 | 0 | 0 | 18 |
| 1 | 1 | 1 | 0 | 0 | 0 | 0 | 10 |
| 0 | 1 | 0 | 1 | 0 | 0 | 0 | 2 |
| 1 | 1 | 1 | 1 | 0 | 0 | 0 | 1 |
| 0 | 1 | 0 | 0 | 1 | 0 | 0 | 3 |
| 0 | 1 | 1 | 0 | 1 | 0 | 0 | 2 |
| 1 | 1 | 1 | 0 | 1 | 0 | 0 | 4 |
| 1 | 1 | 1 | 1 | 1 | 0 | 0 | 1 |
| 0 | 0 | 0 | 0 | 0 | 1 | 0 | 1 |
| 0 | 1 | 0 | 0 | 0 | 1 | 0 | 17 |
| 1 | 0 | 1 | 0 | 0 | 1 | 0 | 1 |
| 0 | 1 | 1 | 0 | 0 | 1 | 0 | 9 |
| 1 | 1 | 1 | 0 | 0 | 1 | 0 | 18 |
| 0 | 0 | 0 | 1 | 0 | 1 | 0 | 1 |
| 0 | 1 | 1 | 1 | 0 | 1 | 0 | 1 |
| 1 | 1 | 1 | 1 | 0 | 1 | 0 | 7 |
| 1 | 0 | 0 | 0 | 1 | 1 | 0 | 1 |
| 0 | 1 | 0 | 0 | 1 | 1 | 0 | 2 |
| 1 | 0 | 1 | 0 | 1 | 1 | 0 | 1 |
| 0 | 1 | 1 | 0 | 1 | 1 | 0 | 1 |
| 1 | 1 | 1 | 0 | 1 | 1 | 0 | 6 |
| 1 | 0 | 1 | 1 | 1 | 1 | 0 | 1 |
| 1 | 1 | 1 | 1 | 1 | 1 | 0 | 6 |
| 0 | 0 | 0 | 0 | 0 | 0 | 1 | 1 |
| 0 | 1 | 0 | 0 | 0 | 0 | 1 | 1 |
| 1 | 1 | 0 | 0 | 0 | 0 | 1 | 1 |
| 0 | 1 | 1 | 0 | 0 | 0 | 1 | 1 |
| 1 | 1 | 1 | 0 | 0 | 0 | 1 | 1 |
| 0 | 1 | 0 | 0 | 1 | 0 | 1 | 1 |
| 0 | 0 | 0 | 0 | 0 | 1 | 1 | 1 |
| 0 | 1 | 1 | 0 | 0 | 1 | 1 | 1 |
| 1 | 1 | 1 | 0 | 0 | 1 | 1 | 4 |
| 0 | 0 | 1 | 1 | 0 | 1 | 1 | 1 |
| 0 | 1 | 1 | 1 | 0 | 1 | 1 | 1 |
| 1 | 1 | 1 | 1 | 0 | 1 | 1 | 2 |
| 1 | 1 | 1 | 0 | 1 | 1 | 1 | 2 |
| 1 | 1 | 1 | 1 | 1 | 1 | 1 | 4 |

**Table S3**: Observed frequencies of allergic bronchopulmonary aspergillosis (ABPA) test patterns estimated by latent class analysis (model 3 with IgE values >1000 IU/mL and high-attenuation mucus) [Af- Aspergillus fumigatus]

| Aspergillus skin test | IgE >1000 IU/mL | Af IgE >0.35 kUA/L | Af precipitins | Total eosinophil count | Central bronchiectasis | Chest radiographic transient opacities | High-attenuation mucus | Observed frequency |
| --- | --- | --- | --- | --- | --- | --- | --- | --- |
| Controlled asthma |  |  |  |  |  |  |  |  |
| 0 | 0 | 0 | 0 | 0 | 0 | 0 | 0 | 17 |
| 1 | 0 | 0 | 0 | 0 | 0 | 0 | 0 | 2 |
| 0 | 1 | 0 | 0 | 0 | 0 | 0 | 0 | 25 |
| 1 | 1 | 0 | 0 | 0 | 0 | 0 | 0 | 4 |
| 0 | 0 | 1 | 0 | 0 | 0 | 0 | 0 | 0 |
| 1 | 0 | 1 | 0 | 0 | 0 | 0 | 0 | 2 |
| 0 | 1 | 1 | 0 | 0 | 0 | 0 | 0 | 15 |
| 1 | 1 | 1 | 0 | 0 | 0 | 0 | 0 | 13 |
| 0 | 0 | 0 | 1 | 0 | 0 | 0 | 0 | 3 |
| 1 | 0 | 0 | 1 | 0 | 0 | 0 | 0 | 0 |
| 0 | 1 | 0 | 1 | 0 | 0 | 0 | 0 | 0 |
| 1 | 1 | 0 | 1 | 0 | 0 | 0 | 0 | 0 |
| 0 | 0 | 1 | 1 | 0 | 0 | 0 | 0 | 0 |
| 1 | 0 | 1 | 1 | 0 | 0 | 0 | 0 | 1 |
| 0 | 1 | 1 | 1 | 0 | 0 | 0 | 0 | 0 |
| 1 | 1 | 1 | 1 | 0 | 0 | 0 | 0 | 0 |
| 0 | 0 | 0 | 0 | 1 | 0 | 0 | 0 | 2 |
| 1 | 0 | 0 | 0 | 1 | 0 | 0 | 0 | 0 |
| 0 | 1 | 0 | 0 | 1 | 0 | 0 | 0 | 2 |
| 1 | 1 | 0 | 0 | 1 | 0 | 0 | 0 | 0 |
| 0 | 0 | 1 | 0 | 1 | 0 | 0 | 0 | 0 |
| 1 | 0 | 1 | 0 | 1 | 0 | 0 | 0 | 0 |
| 0 | 1 | 1 | 0 | 1 | 0 | 0 | 0 | 1 |
| 1 | 1 | 1 | 0 | 1 | 0 | 0 | 0 | 1 |
| 0 | 0 | 0 | 1 | 1 | 0 | 0 | 0 | 0 |
| 1 | 0 | 0 | 1 | 1 | 0 | 0 | 0 | 0 |
| 0 | 1 | 0 | 1 | 1 | 0 | 0 | 0 | 0 |
| 1 | 1 | 0 | 1 | 1 | 0 | 0 | 0 | 0 |
| 0 | 0 | 1 | 1 | 1 | 0 | 0 | 0 | 0 |
| 1 | 0 | 1 | 1 | 1 | 0 | 0 | 0 | 0 |
| 0 | 1 | 1 | 1 | 1 | 0 | 0 | 0 | 0 |
| 1 | 1 | 1 | 1 | 1 | 0 | 0 | 0 | 0 |
| 0 | 0 | 0 | 0 | 0 | 1 | 0 | 0 | 2 |
| 1 | 0 | 0 | 0 | 0 | 1 | 0 | 0 | 1 |
| 0 | 1 | 0 | 0 | 0 | 1 | 0 | 0 | 4 |
| 1 | 1 | 0 | 0 | 0 | 1 | 0 | 0 | 1 |
| 0 | 0 | 1 | 0 | 0 | 1 | 0 | 0 | 1 |
| 1 | 0 | 1 | 0 | 0 | 1 | 0 | 0 | 1 |
| 0 | 1 | 1 | 0 | 0 | 1 | 0 | 0 | 1 |
| 1 | 1 | 1 | 0 | 0 | 1 | 0 | 0 | 10 |
| 0 | 0 | 0 | 1 | 0 | 1 | 0 | 0 | 0 |
| 1 | 0 | 0 | 1 | 0 | 1 | 0 | 0 | 0 |
| 0 | 1 | 0 | 1 | 0 | 1 | 0 | 0 | 0 |
| 1 | 1 | 0 | 1 | 0 | 1 | 0 | 0 | 0 |
| 0 | 0 | 1 | 1 | 0 | 1 | 0 | 0 | 0 |
| 1 | 0 | 1 | 1 | 0 | 1 | 0 | 0 | 0 |
| 0 | 1 | 1 | 1 | 0 | 1 | 0 | 0 | 0 |
| 1 | 1 | 1 | 1 | 0 | 1 | 0 | 0 | 2 |
| 0 | 0 | 0 | 0 | 1 | 1 | 0 | 0 | 0 |
| 1 | 0 | 0 | 0 | 1 | 1 | 0 | 0 | 0 |
| 0 | 1 | 0 | 0 | 1 | 1 | 0 | 0 | 3 |
| 1 | 1 | 0 | 0 | 1 | 1 | 0 | 0 | 0 |
| 0 | 0 | 1 | 0 | 1 | 1 | 0 | 0 | 0 |
| 1 | 0 | 1 | 0 | 1 | 1 | 0 | 0 | 0 |
| 0 | 1 | 1 | 0 | 1 | 1 | 0 | 0 | 0 |
| 1 | 1 | 1 | 0 | 1 | 1 | 0 | 0 | 4 |
| 0 | 0 | 0 | 1 | 1 | 1 | 0 | 0 | 0 |
| 1 | 0 | 0 | 1 | 1 | 1 | 0 | 0 | 0 |
| 0 | 1 | 0 | 1 | 1 | 1 | 0 | 0 | 0 |
| 1 | 1 | 0 | 1 | 1 | 1 | 0 | 0 | 0 |
| 0 | 0 | 1 | 1 | 1 | 1 | 0 | 0 | 0 |
| 1 | 0 | 1 | 1 | 1 | 1 | 0 | 0 | 0 |
| 0 | 1 | 1 | 1 | 1 | 1 | 0 | 0 | 0 |
| 1 | 1 | 1 | 1 | 1 | 1 | 0 | 0 | 0 |
| 0 | 0 | 0 | 0 | 0 | 0 | 1 | 0 | 0 |
| 1 | 0 | 0 | 0 | 0 | 0 | 1 | 0 | 1 |
| 0 | 1 | 0 | 0 | 0 | 0 | 1 | 0 | 0 |
| 1 | 1 | 0 | 0 | 0 | 0 | 1 | 0 | 0 |
| 0 | 0 | 1 | 0 | 0 | 0 | 1 | 0 | 0 |
| 1 | 0 | 1 | 0 | 0 | 0 | 1 | 0 | 0 |
| 0 | 1 | 1 | 0 | 0 | 0 | 1 | 0 | 1 |
| 1 | 1 | 1 | 0 | 0 | 0 | 1 | 0 | 0 |
| 0 | 0 | 0 | 1 | 0 | 0 | 1 | 0 | 0 |
| 1 | 0 | 0 | 1 | 0 | 0 | 1 | 0 | 0 |
| 0 | 1 | 0 | 1 | 0 | 0 | 1 | 0 | 0 |
| 1 | 1 | 0 | 1 | 0 | 0 | 1 | 0 | 0 |
| 0 | 0 | 1 | 1 | 0 | 0 | 1 | 0 | 0 |
| 1 | 0 | 1 | 1 | 0 | 0 | 1 | 0 | 0 |
| 0 | 1 | 1 | 1 | 0 | 0 | 1 | 0 | 0 |
| 1 | 1 | 1 | 1 | 0 | 0 | 1 | 0 | 0 |
| 0 | 0 | 0 | 0 | 1 | 0 | 1 | 0 | 0 |
| 1 | 0 | 0 | 0 | 1 | 0 | 1 | 0 | 0 |
| 0 | 1 | 0 | 0 | 1 | 0 | 1 | 0 | 0 |
| 1 | 1 | 0 | 0 | 1 | 0 | 1 | 0 | 0 |
| 0 | 0 | 1 | 0 | 1 | 0 | 1 | 0 | 0 |
| 1 | 0 | 1 | 0 | 1 | 0 | 1 | 0 | 0 |
| 0 | 1 | 1 | 0 | 1 | 0 | 1 | 0 | 0 |
| 1 | 1 | 1 | 0 | 1 | 0 | 1 | 0 | 0 |
| 0 | 0 | 0 | 1 | 1 | 0 | 1 | 0 | 0 |
| 1 | 0 | 0 | 1 | 1 | 0 | 1 | 0 | 0 |
| 0 | 1 | 0 | 1 | 1 | 0 | 1 | 0 | 0 |
| 1 | 1 | 0 | 1 | 1 | 0 | 1 | 0 | 0 |
| 0 | 0 | 1 | 1 | 1 | 0 | 1 | 0 | 0 |
| 1 | 0 | 1 | 1 | 1 | 0 | 1 | 0 | 0 |
| 0 | 1 | 1 | 1 | 1 | 0 | 1 | 0 | 0 |
| 1 | 1 | 1 | 1 | 1 | 0 | 1 | 0 | 0 |
| 0 | 0 | 0 | 0 | 0 | 1 | 1 | 0 | 1 |
| 1 | 0 | 0 | 0 | 0 | 1 | 1 | 0 | 0 |
| 0 | 1 | 0 | 0 | 0 | 1 | 1 | 0 | 0 |
| 1 | 1 | 0 | 0 | 0 | 1 | 1 | 0 | 0 |
| 0 | 0 | 1 | 0 | 0 | 1 | 1 | 0 | 0 |
| 1 | 0 | 1 | 0 | 0 | 1 | 1 | 0 | 0 |
| 0 | 1 | 1 | 0 | 0 | 1 | 1 | 0 | 0 |
| 1 | 1 | 1 | 0 | 0 | 1 | 1 | 0 | 1 |
| 0 | 0 | 0 | 1 | 0 | 1 | 1 | 0 | 0 |
| 1 | 0 | 0 | 1 | 0 | 1 | 1 | 0 | 0 |
| 0 | 1 | 0 | 1 | 0 | 1 | 1 | 0 | 0 |
| 1 | 1 | 0 | 1 | 0 | 1 | 1 | 0 | 0 |
| 0 | 0 | 1 | 1 | 0 | 1 | 1 | 0 | 0 |
| 1 | 0 | 1 | 1 | 0 | 1 | 1 | 0 | 0 |
| 0 | 1 | 1 | 1 | 0 | 1 | 1 | 0 | 1 |
| 1 | 1 | 1 | 1 | 0 | 1 | 1 | 0 | 3 |
| 0 | 0 | 0 | 0 | 1 | 1 | 1 | 0 | 0 |
| 1 | 0 | 0 | 0 | 1 | 1 | 1 | 0 | 0 |
| 0 | 1 | 0 | 0 | 1 | 1 | 1 | 0 | 0 |
| 1 | 1 | 0 | 0 | 1 | 1 | 1 | 0 | 0 |
| 0 | 0 | 1 | 0 | 1 | 1 | 1 | 0 | 0 |
| 1 | 0 | 1 | 0 | 1 | 1 | 1 | 0 | 0 |
| 0 | 1 | 1 | 0 | 1 | 1 | 1 | 0 | 1 |
| 1 | 1 | 1 | 0 | 1 | 1 | 1 | 0 | 0 |
| 0 | 0 | 0 | 1 | 1 | 1 | 1 | 0 | 0 |
| 1 | 0 | 0 | 1 | 1 | 1 | 1 | 0 | 0 |
| 0 | 1 | 0 | 1 | 1 | 1 | 1 | 0 | 0 |
| 1 | 1 | 0 | 1 | 1 | 1 | 1 | 0 | 0 |
| 0 | 0 | 1 | 1 | 1 | 1 | 1 | 0 | 0 |
| 1 | 0 | 1 | 1 | 1 | 1 | 1 | 0 | 0 |
| 0 | 1 | 1 | 1 | 1 | 1 | 1 | 0 | 0 |
| 1 | 1 | 1 | 1 | 1 | 1 | 1 | 0 | 1 |
| 0 | 0 | 0 | 0 | 0 | 0 | 0 | 1 | 0 |
| 1 | 0 | 0 | 0 | 0 | 0 | 0 | 1 | 0 |
| 0 | 1 | 0 | 0 | 0 | 0 | 0 | 1 | 0 |
| 1 | 1 | 0 | 0 | 0 | 0 | 0 | 1 | 0 |
| 0 | 0 | 1 | 0 | 0 | 0 | 0 | 1 | 0 |
| 1 | 0 | 1 | 0 | 0 | 0 | 0 | 1 | 0 |
| 0 | 1 | 1 | 0 | 0 | 0 | 0 | 1 | 0 |
| 1 | 1 | 1 | 0 | 0 | 0 | 0 | 1 | 0 |
| 0 | 0 | 0 | 1 | 0 | 0 | 0 | 1 | 0 |
| 1 | 0 | 0 | 1 | 0 | 0 | 0 | 1 | 0 |
| 0 | 1 | 0 | 1 | 0 | 0 | 0 | 1 | 0 |
| 1 | 1 | 0 | 1 | 0 | 0 | 0 | 1 | 0 |
| 0 | 0 | 1 | 1 | 0 | 0 | 0 | 1 | 0 |
| 1 | 0 | 1 | 1 | 0 | 0 | 0 | 1 | 0 |
| 0 | 1 | 1 | 1 | 0 | 0 | 0 | 1 | 0 |
| 1 | 1 | 1 | 1 | 0 | 0 | 0 | 1 | 0 |
| 0 | 0 | 0 | 0 | 1 | 0 | 0 | 1 | 0 |
| 1 | 0 | 0 | 0 | 1 | 0 | 0 | 1 | 0 |
| 0 | 1 | 0 | 0 | 1 | 0 | 0 | 1 | 0 |
| 1 | 1 | 0 | 0 | 1 | 0 | 0 | 1 | 0 |
| 0 | 0 | 1 | 0 | 1 | 0 | 0 | 1 | 0 |
| 1 | 0 | 1 | 0 | 1 | 0 | 0 | 1 | 0 |
| 0 | 1 | 1 | 0 | 1 | 0 | 0 | 1 | 0 |
| 1 | 1 | 1 | 0 | 1 | 0 | 0 | 1 | 0 |
| 0 | 0 | 0 | 1 | 1 | 0 | 0 | 1 | 0 |
| 1 | 0 | 0 | 1 | 1 | 0 | 0 | 1 | 0 |
| 0 | 1 | 0 | 1 | 1 | 0 | 0 | 1 | 0 |
| 1 | 1 | 0 | 1 | 1 | 0 | 0 | 1 | 0 |
| 0 | 0 | 1 | 1 | 1 | 0 | 0 | 1 | 0 |
| 1 | 0 | 1 | 1 | 1 | 0 | 0 | 1 | 0 |
| 0 | 1 | 1 | 1 | 1 | 0 | 0 | 1 | 0 |
| 1 | 1 | 1 | 1 | 1 | 0 | 0 | 1 | 0 |
| 0 | 0 | 0 | 0 | 0 | 1 | 0 | 1 | 0 |
| 1 | 0 | 0 | 0 | 0 | 1 | 0 | 1 | 0 |
| 0 | 1 | 0 | 0 | 0 | 1 | 0 | 1 | 0 |
| 1 | 1 | 0 | 0 | 0 | 1 | 0 | 1 | 0 |
| 0 | 0 | 1 | 0 | 0 | 1 | 0 | 1 | 0 |
| 1 | 0 | 1 | 0 | 0 | 1 | 0 | 1 | 0 |
| 0 | 1 | 1 | 0 | 0 | 1 | 0 | 1 | 0 |
| 1 | 1 | 1 | 0 | 0 | 1 | 0 | 1 | 0 |
| 0 | 0 | 0 | 1 | 0 | 1 | 0 | 1 | 0 |
| 1 | 0 | 0 | 1 | 0 | 1 | 0 | 1 | 0 |
| 0 | 1 | 0 | 1 | 0 | 1 | 0 | 1 | 0 |
| 1 | 1 | 0 | 1 | 0 | 1 | 0 | 1 | 0 |
| 0 | 0 | 1 | 1 | 0 | 1 | 0 | 1 | 0 |
| 1 | 0 | 1 | 1 | 0 | 1 | 0 | 1 | 0 |
| 0 | 1 | 1 | 1 | 0 | 1 | 0 | 1 | 0 |
| 1 | 1 | 1 | 1 | 0 | 1 | 0 | 1 | 4 |
| 0 | 0 | 0 | 0 | 1 | 1 | 0 | 1 | 0 |
| 1 | 0 | 0 | 0 | 1 | 1 | 0 | 1 | 0 |
| 0 | 1 | 0 | 0 | 1 | 1 | 0 | 1 | 0 |
| 1 | 1 | 0 | 0 | 1 | 1 | 0 | 1 | 0 |
| 0 | 0 | 1 | 0 | 1 | 1 | 0 | 1 | 0 |
| 1 | 0 | 1 | 0 | 1 | 1 | 0 | 1 | 0 |
| 0 | 1 | 1 | 0 | 1 | 1 | 0 | 1 | 0 |
| 1 | 1 | 1 | 0 | 1 | 1 | 0 | 1 | 0 |
| 0 | 0 | 0 | 1 | 1 | 1 | 0 | 1 | 0 |
| 1 | 0 | 0 | 1 | 1 | 1 | 0 | 1 | 0 |
| 0 | 1 | 0 | 1 | 1 | 1 | 0 | 1 | 0 |
| 1 | 1 | 0 | 1 | 1 | 1 | 0 | 1 | 0 |
| 0 | 0 | 1 | 1 | 1 | 1 | 0 | 1 | 0 |
| 1 | 0 | 1 | 1 | 1 | 1 | 0 | 1 | 0 |
| 0 | 1 | 1 | 1 | 1 | 1 | 0 | 1 | 0 |
| 1 | 1 | 1 | 1 | 1 | 1 | 0 | 1 | 1 |
| 0 | 0 | 0 | 0 | 0 | 0 | 1 | 1 | 0 |
| 1 | 0 | 0 | 0 | 0 | 0 | 1 | 1 | 0 |
| 0 | 1 | 0 | 0 | 0 | 0 | 1 | 1 | 0 |
| 1 | 1 | 0 | 0 | 0 | 0 | 1 | 1 | 0 |
| 0 | 0 | 1 | 0 | 0 | 0 | 1 | 1 | 0 |
| 1 | 0 | 1 | 0 | 0 | 0 | 1 | 1 | 0 |
| 0 | 1 | 1 | 0 | 0 | 0 | 1 | 1 | 0 |
| 1 | 1 | 1 | 0 | 0 | 0 | 1 | 1 | 0 |
| 0 | 0 | 0 | 1 | 0 | 0 | 1 | 1 | 0 |
| 1 | 0 | 0 | 1 | 0 | 0 | 1 | 1 | 0 |
| 0 | 1 | 0 | 1 | 0 | 0 | 1 | 1 | 0 |
| 1 | 1 | 0 | 1 | 0 | 0 | 1 | 1 | 0 |
| 0 | 0 | 1 | 1 | 0 | 0 | 1 | 1 | 0 |
| 1 | 0 | 1 | 1 | 0 | 0 | 1 | 1 | 0 |
| 0 | 1 | 1 | 1 | 0 | 0 | 1 | 1 | 0 |
| 1 | 1 | 1 | 1 | 0 | 0 | 1 | 1 | 0 |
| 0 | 0 | 0 | 0 | 1 | 0 | 1 | 1 | 0 |
| 1 | 0 | 0 | 0 | 1 | 0 | 1 | 1 | 0 |
| 0 | 1 | 0 | 0 | 1 | 0 | 1 | 1 | 0 |
| 1 | 1 | 0 | 0 | 1 | 0 | 1 | 1 | 0 |
| 0 | 0 | 1 | 0 | 1 | 0 | 1 | 1 | 0 |
| 1 | 0 | 1 | 0 | 1 | 0 | 1 | 1 | 0 |
| 0 | 1 | 1 | 0 | 1 | 0 | 1 | 1 | 0 |
| 1 | 1 | 1 | 0 | 1 | 0 | 1 | 1 | 0 |
| 0 | 0 | 0 | 1 | 1 | 0 | 1 | 1 | 0 |
| 1 | 0 | 0 | 1 | 1 | 0 | 1 | 1 | 0 |
| 0 | 1 | 0 | 1 | 1 | 0 | 1 | 1 | 0 |
| 1 | 1 | 0 | 1 | 1 | 0 | 1 | 1 | 0 |
| 0 | 0 | 1 | 1 | 1 | 0 | 1 | 1 | 0 |
| 1 | 0 | 1 | 1 | 1 | 0 | 1 | 1 | 0 |
| 0 | 1 | 1 | 1 | 1 | 0 | 1 | 1 | 0 |
| 1 | 1 | 1 | 1 | 1 | 0 | 1 | 1 | 0 |
| 0 | 0 | 0 | 0 | 0 | 1 | 1 | 1 | 0 |
| 1 | 0 | 0 | 0 | 0 | 1 | 1 | 1 | 0 |
| 0 | 1 | 0 | 0 | 0 | 1 | 1 | 1 | 0 |
| 1 | 1 | 0 | 0 | 0 | 1 | 1 | 1 | 0 |
| 0 | 0 | 1 | 0 | 0 | 1 | 1 | 1 | 0 |
| 1 | 0 | 1 | 0 | 0 | 1 | 1 | 1 | 0 |
| 0 | 1 | 1 | 0 | 0 | 1 | 1 | 1 | 0 |
| 1 | 1 | 1 | 0 | 0 | 1 | 1 | 1 | 1 |
| 0 | 0 | 0 | 1 | 0 | 1 | 1 | 1 | 0 |
| 1 | 0 | 0 | 1 | 0 | 1 | 1 | 1 | 0 |
| 0 | 1 | 0 | 1 | 0 | 1 | 1 | 1 | 0 |
| 1 | 1 | 0 | 1 | 0 | 1 | 1 | 1 | 0 |
| 0 | 0 | 1 | 1 | 0 | 1 | 1 | 1 | 0 |
| 1 | 0 | 1 | 1 | 0 | 1 | 1 | 1 | 0 |
| 0 | 1 | 1 | 1 | 0 | 1 | 1 | 1 | 0 |
| 1 | 1 | 1 | 1 | 0 | 1 | 1 | 1 | 1 |
| 0 | 0 | 0 | 0 | 1 | 1 | 1 | 1 | 0 |
| 1 | 0 | 0 | 0 | 1 | 1 | 1 | 1 | 0 |
| 0 | 1 | 0 | 0 | 1 | 1 | 1 | 1 | 0 |
| 1 | 1 | 0 | 0 | 1 | 1 | 1 | 1 | 0 |
| 0 | 0 | 1 | 0 | 1 | 1 | 1 | 1 | 0 |
| 1 | 0 | 1 | 0 | 1 | 1 | 1 | 1 | 0 |
| 0 | 1 | 1 | 0 | 1 | 1 | 1 | 1 | 0 |
| 1 | 1 | 1 | 0 | 1 | 1 | 1 | 1 | 0 |
| 0 | 0 | 0 | 1 | 1 | 1 | 1 | 1 | 0 |
| 1 | 0 | 0 | 1 | 1 | 1 | 1 | 1 | 0 |
| 0 | 1 | 0 | 1 | 1 | 1 | 1 | 1 | 0 |
| 1 | 1 | 0 | 1 | 1 | 1 | 1 | 1 | 0 |
| 0 | 0 | 1 | 1 | 1 | 1 | 1 | 1 | 0 |
| 1 | 0 | 1 | 1 | 1 | 1 | 1 | 1 | 0 |
| 0 | 1 | 1 | 1 | 1 | 1 | 1 | 1 | 0 |
| 1 | 1 | 1 | 1 | 1 | 1 | 1 | 1 | 2 |
| Uncontrolled asthma |  |  |  |  |  |  |  |  |
| 0 | 0 | 0 | 0 | 0 | 0 | 0 | 0 | 40 |
| 1 | 0 | 0 | 0 | 0 | 0 | 0 | 0 | 9 |
| 0 | 1 | 0 | 0 | 0 | 0 | 0 | 0 | 45 |
| 1 | 1 | 0 | 0 | 0 | 0 | 0 | 0 | 2 |
| 0 | 0 | 1 | 0 | 0 | 0 | 0 | 0 | 3 |
| 1 | 0 | 1 | 0 | 0 | 0 | 0 | 0 | 4 |
| 0 | 1 | 1 | 0 | 0 | 0 | 0 | 0 | 16 |
| 1 | 1 | 1 | 0 | 0 | 0 | 0 | 0 | 8 |
| 0 | 0 | 0 | 1 | 0 | 0 | 0 | 0 | 1 |
| 1 | 0 | 0 | 1 | 0 | 0 | 0 | 0 | 0 |
| 0 | 1 | 0 | 1 | 0 | 0 | 0 | 0 | 1 |
| 1 | 1 | 0 | 1 | 0 | 0 | 0 | 0 | 0 |
| 0 | 0 | 1 | 1 | 0 | 0 | 0 | 0 | 1 |
| 1 | 0 | 1 | 1 | 0 | 0 | 0 | 0 | 0 |
| 0 | 1 | 1 | 1 | 0 | 0 | 0 | 0 | 0 |
| 1 | 1 | 1 | 1 | 0 | 0 | 0 | 0 | 1 |
| 0 | 0 | 0 | 0 | 1 | 0 | 0 | 0 | 1 |
| 1 | 0 | 0 | 0 | 1 | 0 | 0 | 0 | 0 |
| 0 | 1 | 0 | 0 | 1 | 0 | 0 | 0 | 2 |
| 1 | 1 | 0 | 0 | 1 | 0 | 0 | 0 | 0 |
| 0 | 0 | 1 | 0 | 1 | 0 | 0 | 0 | 0 |
| 1 | 0 | 1 | 0 | 1 | 0 | 0 | 0 | 1 |
| 0 | 1 | 1 | 0 | 1 | 0 | 0 | 0 | 2 |
| 1 | 1 | 1 | 0 | 1 | 0 | 0 | 0 | 3 |
| 0 | 0 | 0 | 1 | 1 | 0 | 0 | 0 | 0 |
| 1 | 0 | 0 | 1 | 1 | 0 | 0 | 0 | 0 |
| 0 | 1 | 0 | 1 | 1 | 0 | 0 | 0 | 0 |
| 1 | 1 | 0 | 1 | 1 | 0 | 0 | 0 | 0 |
| 0 | 0 | 1 | 1 | 1 | 0 | 0 | 0 | 0 |
| 1 | 0 | 1 | 1 | 1 | 0 | 0 | 0 | 0 |
| 0 | 1 | 1 | 1 | 1 | 0 | 0 | 0 | 0 |
| 1 | 1 | 1 | 1 | 1 | 0 | 0 | 0 | 1 |
| 0 | 0 | 0 | 0 | 0 | 1 | 0 | 0 | 4 |
| 1 | 0 | 0 | 0 | 0 | 1 | 0 | 0 | 0 |
| 0 | 1 | 0 | 0 | 0 | 1 | 0 | 0 | 14 |
| 1 | 1 | 0 | 0 | 0 | 1 | 0 | 0 | 1 |
| 0 | 0 | 1 | 0 | 0 | 1 | 0 | 0 | 1 |
| 1 | 0 | 1 | 0 | 0 | 1 | 0 | 0 | 2 |
| 0 | 1 | 1 | 0 | 0 | 1 | 0 | 0 | 8 |
| 1 | 1 | 1 | 0 | 0 | 1 | 0 | 0 | 17 |
| 0 | 0 | 0 | 1 | 0 | 1 | 0 | 0 | 1 |
| 1 | 0 | 0 | 1 | 0 | 1 | 0 | 0 | 0 |
| 0 | 1 | 0 | 1 | 0 | 1 | 0 | 0 | 0 |
| 1 | 1 | 0 | 1 | 0 | 1 | 0 | 0 | 0 |
| 0 | 0 | 1 | 1 | 0 | 1 | 0 | 0 | 0 |
| 1 | 0 | 1 | 1 | 0 | 1 | 0 | 0 | 1 |
| 0 | 1 | 1 | 1 | 0 | 1 | 0 | 0 | 0 |
| 1 | 1 | 1 | 1 | 0 | 1 | 0 | 0 | 2 |
| 0 | 0 | 0 | 0 | 1 | 1 | 0 | 0 | 2 |
| 1 | 0 | 0 | 0 | 1 | 1 | 0 | 0 | 1 |
| 0 | 1 | 0 | 0 | 1 | 1 | 0 | 0 | 0 |
| 1 | 1 | 0 | 0 | 1 | 1 | 0 | 0 | 0 |
| 0 | 0 | 1 | 0 | 1 | 1 | 0 | 0 | 0 |
| 1 | 0 | 1 | 0 | 1 | 1 | 0 | 0 | 1 |
| 0 | 1 | 1 | 0 | 1 | 1 | 0 | 0 | 1 |
| 1 | 1 | 1 | 0 | 1 | 1 | 0 | 0 | 0 |
| 0 | 0 | 0 | 1 | 1 | 1 | 0 | 0 | 0 |
| 1 | 0 | 0 | 1 | 1 | 1 | 0 | 0 | 0 |
| 0 | 1 | 0 | 1 | 1 | 1 | 0 | 0 | 0 |
| 1 | 1 | 0 | 1 | 1 | 1 | 0 | 0 | 0 |
| 0 | 0 | 1 | 1 | 1 | 1 | 0 | 0 | 0 |
| 1 | 0 | 1 | 1 | 1 | 1 | 0 | 0 | 0 |
| 0 | 1 | 1 | 1 | 1 | 1 | 0 | 0 | 0 |
| 1 | 1 | 1 | 1 | 1 | 1 | 0 | 0 | 1 |
| 0 | 0 | 0 | 0 | 0 | 0 | 1 | 0 | 1 |
| 1 | 0 | 0 | 0 | 0 | 0 | 1 | 0 | 0 |
| 0 | 1 | 0 | 0 | 0 | 0 | 1 | 0 | 1 |
| 1 | 1 | 0 | 0 | 0 | 0 | 1 | 0 | 0 |
| 0 | 0 | 1 | 0 | 0 | 0 | 1 | 0 | 0 |
| 1 | 0 | 1 | 0 | 0 | 0 | 1 | 0 | 0 |
| 0 | 1 | 1 | 0 | 0 | 0 | 1 | 0 | 1 |
| 1 | 1 | 1 | 0 | 0 | 0 | 1 | 0 | 1 |
| 0 | 0 | 0 | 1 | 0 | 0 | 1 | 0 | 0 |
| 1 | 0 | 0 | 1 | 0 | 0 | 1 | 0 | 0 |
| 0 | 1 | 0 | 1 | 0 | 0 | 1 | 0 | 0 |
| 1 | 1 | 0 | 1 | 0 | 0 | 1 | 0 | 0 |
| 0 | 0 | 1 | 1 | 0 | 0 | 1 | 0 | 0 |
| 1 | 0 | 1 | 1 | 0 | 0 | 1 | 0 | 0 |
| 0 | 1 | 1 | 1 | 0 | 0 | 1 | 0 | 0 |
| 1 | 1 | 1 | 1 | 0 | 0 | 1 | 0 | 0 |
| 0 | 0 | 0 | 0 | 1 | 0 | 1 | 0 | 0 |
| 1 | 0 | 0 | 0 | 1 | 0 | 1 | 0 | 0 |
| 0 | 1 | 0 | 0 | 1 | 0 | 1 | 0 | 1 |
| 1 | 1 | 0 | 0 | 1 | 0 | 1 | 0 | 0 |
| 0 | 0 | 1 | 0 | 1 | 0 | 1 | 0 | 0 |
| 1 | 0 | 1 | 0 | 1 | 0 | 1 | 0 | 0 |
| 0 | 1 | 1 | 0 | 1 | 0 | 1 | 0 | 0 |
| 1 | 1 | 1 | 0 | 1 | 0 | 1 | 0 | 0 |
| 0 | 0 | 0 | 1 | 1 | 0 | 1 | 0 | 0 |
| 1 | 0 | 0 | 1 | 1 | 0 | 1 | 0 | 0 |
| 0 | 1 | 0 | 1 | 1 | 0 | 1 | 0 | 0 |
| 1 | 1 | 0 | 1 | 1 | 0 | 1 | 0 | 0 |
| 0 | 0 | 1 | 1 | 1 | 0 | 1 | 0 | 0 |
| 1 | 0 | 1 | 1 | 1 | 0 | 1 | 0 | 0 |
| 0 | 1 | 1 | 1 | 1 | 0 | 1 | 0 | 0 |
| 1 | 1 | 1 | 1 | 1 | 0 | 1 | 0 | 0 |
| 0 | 0 | 0 | 0 | 0 | 1 | 1 | 0 | 1 |
| 1 | 0 | 0 | 0 | 0 | 1 | 1 | 0 | 0 |
| 0 | 1 | 0 | 0 | 0 | 1 | 1 | 0 | 0 |
| 1 | 1 | 0 | 0 | 0 | 1 | 1 | 0 | 0 |
| 0 | 0 | 1 | 0 | 0 | 1 | 1 | 0 | 0 |
| 1 | 0 | 1 | 0 | 0 | 1 | 1 | 0 | 0 |
| 0 | 1 | 1 | 0 | 0 | 1 | 1 | 0 | 1 |
| 1 | 1 | 1 | 0 | 0 | 1 | 1 | 0 | 2 |
| 0 | 0 | 0 | 1 | 0 | 1 | 1 | 0 | 0 |
| 1 | 0 | 0 | 1 | 0 | 1 | 1 | 0 | 0 |
| 0 | 1 | 0 | 1 | 0 | 1 | 1 | 0 | 0 |
| 1 | 1 | 0 | 1 | 0 | 1 | 1 | 0 | 0 |
| 0 | 0 | 1 | 1 | 0 | 1 | 1 | 0 | 0 |
| 1 | 0 | 1 | 1 | 0 | 1 | 1 | 0 | 0 |
| 0 | 1 | 1 | 1 | 0 | 1 | 1 | 0 | 0 |
| 1 | 1 | 1 | 1 | 0 | 1 | 1 | 0 | 1 |
| 0 | 0 | 0 | 0 | 1 | 1 | 1 | 0 | 0 |
| 1 | 0 | 0 | 0 | 1 | 1 | 1 | 0 | 0 |
| 0 | 1 | 0 | 0 | 1 | 1 | 1 | 0 | 0 |
| 1 | 1 | 0 | 0 | 1 | 1 | 1 | 0 | 0 |
| 0 | 0 | 1 | 0 | 1 | 1 | 1 | 0 | 0 |
| 1 | 0 | 1 | 0 | 1 | 1 | 1 | 0 | 0 |
| 0 | 1 | 1 | 0 | 1 | 1 | 1 | 0 | 0 |
| 1 | 1 | 1 | 0 | 1 | 1 | 1 | 0 | 0 |
| 0 | 0 | 0 | 1 | 1 | 1 | 1 | 0 | 0 |
| 1 | 0 | 0 | 1 | 1 | 1 | 1 | 0 | 0 |
| 0 | 1 | 0 | 1 | 1 | 1 | 1 | 0 | 0 |
| 1 | 1 | 0 | 1 | 1 | 1 | 1 | 0 | 0 |
| 0 | 0 | 1 | 1 | 1 | 1 | 1 | 0 | 0 |
| 1 | 0 | 1 | 1 | 1 | 1 | 1 | 0 | 0 |
| 0 | 1 | 1 | 1 | 1 | 1 | 1 | 0 | 0 |
| 1 | 1 | 1 | 1 | 1 | 1 | 1 | 0 | 0 |
| 0 | 0 | 0 | 0 | 0 | 0 | 0 | 1 | 0 |
| 1 | 0 | 0 | 0 | 0 | 0 | 0 | 1 | 0 |
| 0 | 1 | 0 | 0 | 0 | 0 | 0 | 1 | 0 |
| 1 | 1 | 0 | 0 | 0 | 0 | 0 | 1 | 0 |
| 0 | 0 | 1 | 0 | 0 | 0 | 0 | 1 | 0 |
| 1 | 0 | 1 | 0 | 0 | 0 | 0 | 1 | 0 |
| 0 | 1 | 1 | 0 | 0 | 0 | 0 | 1 | 0 |
| 1 | 1 | 1 | 0 | 0 | 0 | 0 | 1 | 0 |
| 0 | 0 | 0 | 1 | 0 | 0 | 0 | 1 | 0 |
| 1 | 0 | 0 | 1 | 0 | 0 | 0 | 1 | 0 |
| 0 | 1 | 0 | 1 | 0 | 0 | 0 | 1 | 0 |
| 1 | 1 | 0 | 1 | 0 | 0 | 0 | 1 | 0 |
| 0 | 0 | 1 | 1 | 0 | 0 | 0 | 1 | 0 |
| 1 | 0 | 1 | 1 | 0 | 0 | 0 | 1 | 0 |
| 0 | 1 | 1 | 1 | 0 | 0 | 0 | 1 | 0 |
| 1 | 1 | 1 | 1 | 0 | 0 | 0 | 1 | 0 |
| 0 | 0 | 0 | 0 | 1 | 0 | 0 | 1 | 0 |
| 1 | 0 | 0 | 0 | 1 | 0 | 0 | 1 | 0 |
| 0 | 1 | 0 | 0 | 1 | 0 | 0 | 1 | 0 |
| 1 | 1 | 0 | 0 | 1 | 0 | 0 | 1 | 0 |
| 0 | 0 | 1 | 0 | 1 | 0 | 0 | 1 | 0 |
| 1 | 0 | 1 | 0 | 1 | 0 | 0 | 1 | 0 |
| 0 | 1 | 1 | 0 | 1 | 0 | 0 | 1 | 0 |
| 1 | 1 | 1 | 0 | 1 | 0 | 0 | 1 | 0 |
| 0 | 0 | 0 | 1 | 1 | 0 | 0 | 1 | 0 |
| 1 | 0 | 0 | 1 | 1 | 0 | 0 | 1 | 0 |
| 0 | 1 | 0 | 1 | 1 | 0 | 0 | 1 | 0 |
| 1 | 1 | 0 | 1 | 1 | 0 | 0 | 1 | 0 |
| 0 | 0 | 1 | 1 | 1 | 0 | 0 | 1 | 0 |
| 1 | 0 | 1 | 1 | 1 | 0 | 0 | 1 | 0 |
| 0 | 1 | 1 | 1 | 1 | 0 | 0 | 1 | 0 |
| 1 | 1 | 1 | 1 | 1 | 0 | 0 | 1 | 0 |
| 0 | 0 | 0 | 0 | 0 | 1 | 0 | 1 | 0 |
| 1 | 0 | 0 | 0 | 0 | 1 | 0 | 1 | 0 |
| 0 | 1 | 0 | 0 | 0 | 1 | 0 | 1 | 0 |
| 1 | 1 | 0 | 0 | 0 | 1 | 0 | 1 | 0 |
| 0 | 0 | 1 | 0 | 0 | 1 | 0 | 1 | 0 |
| 1 | 0 | 1 | 0 | 0 | 1 | 0 | 1 | 0 |
| 0 | 1 | 1 | 0 | 0 | 1 | 0 | 1 | 0 |
| 1 | 1 | 1 | 0 | 0 | 1 | 0 | 1 | 0 |
| 0 | 0 | 0 | 1 | 0 | 1 | 0 | 1 | 0 |
| 1 | 0 | 0 | 1 | 0 | 1 | 0 | 1 | 0 |
| 0 | 1 | 0 | 1 | 0 | 1 | 0 | 1 | 0 |
| 1 | 1 | 0 | 1 | 0 | 1 | 0 | 1 | 0 |
| 0 | 0 | 1 | 1 | 0 | 1 | 0 | 1 | 0 |
| 1 | 0 | 1 | 1 | 0 | 1 | 0 | 1 | 0 |
| 0 | 1 | 1 | 1 | 0 | 1 | 0 | 1 | 0 |
| 1 | 1 | 1 | 1 | 0 | 1 | 0 | 1 | 4 |
| 0 | 0 | 0 | 0 | 1 | 1 | 0 | 1 | 0 |
| 1 | 0 | 0 | 0 | 1 | 1 | 0 | 1 | 0 |
| 0 | 1 | 0 | 0 | 1 | 1 | 0 | 1 | 0 |
| 1 | 1 | 0 | 0 | 1 | 1 | 0 | 1 | 0 |
| 0 | 0 | 1 | 0 | 1 | 1 | 0 | 1 | 0 |
| 1 | 0 | 1 | 0 | 1 | 1 | 0 | 1 | 0 |
| 0 | 1 | 1 | 0 | 1 | 1 | 0 | 1 | 0 |
| 1 | 1 | 1 | 0 | 1 | 1 | 0 | 1 | 6 |
| 0 | 0 | 0 | 1 | 1 | 1 | 0 | 1 | 0 |
| 1 | 0 | 0 | 1 | 1 | 1 | 0 | 1 | 0 |
| 0 | 1 | 0 | 1 | 1 | 1 | 0 | 1 | 0 |
| 1 | 1 | 0 | 1 | 1 | 1 | 0 | 1 | 0 |
| 0 | 0 | 1 | 1 | 1 | 1 | 0 | 1 | 0 |
| 1 | 0 | 1 | 1 | 1 | 1 | 0 | 1 | 0 |
| 0 | 1 | 1 | 1 | 1 | 1 | 0 | 1 | 0 |
| 1 | 1 | 1 | 1 | 1 | 1 | 0 | 1 | 6 |
| 0 | 0 | 0 | 0 | 0 | 0 | 1 | 1 | 0 |
| 1 | 0 | 0 | 0 | 0 | 0 | 1 | 1 | 0 |
| 0 | 1 | 0 | 0 | 0 | 0 | 1 | 1 | 0 |
| 1 | 1 | 0 | 0 | 0 | 0 | 1 | 1 | 0 |
| 0 | 0 | 1 | 0 | 0 | 0 | 1 | 1 | 0 |
| 1 | 0 | 1 | 0 | 0 | 0 | 1 | 1 | 0 |
| 0 | 1 | 1 | 0 | 0 | 0 | 1 | 1 | 0 |
| 1 | 1 | 1 | 0 | 0 | 0 | 1 | 1 | 0 |
| 0 | 0 | 0 | 1 | 0 | 0 | 1 | 1 | 0 |
| 1 | 0 | 0 | 1 | 0 | 0 | 1 | 1 | 0 |
| 0 | 1 | 0 | 1 | 0 | 0 | 1 | 1 | 0 |
| 1 | 1 | 0 | 1 | 0 | 0 | 1 | 1 | 0 |
| 0 | 0 | 1 | 1 | 0 | 0 | 1 | 1 | 0 |
| 1 | 0 | 1 | 1 | 0 | 0 | 1 | 1 | 0 |
| 0 | 1 | 1 | 1 | 0 | 0 | 1 | 1 | 0 |
| 1 | 1 | 1 | 1 | 0 | 0 | 1 | 1 | 0 |
| 0 | 0 | 0 | 0 | 1 | 0 | 1 | 1 | 0 |
| 1 | 0 | 0 | 0 | 1 | 0 | 1 | 1 | 0 |
| 0 | 1 | 0 | 0 | 1 | 0 | 1 | 1 | 0 |
| 1 | 1 | 0 | 0 | 1 | 0 | 1 | 1 | 0 |
| 0 | 0 | 1 | 0 | 1 | 0 | 1 | 1 | 0 |
| 1 | 0 | 1 | 0 | 1 | 0 | 1 | 1 | 0 |
| 0 | 1 | 1 | 0 | 1 | 0 | 1 | 1 | 0 |
| 1 | 1 | 1 | 0 | 1 | 0 | 1 | 1 | 0 |
| 0 | 0 | 0 | 1 | 1 | 0 | 1 | 1 | 0 |
| 1 | 0 | 0 | 1 | 1 | 0 | 1 | 1 | 0 |
| 0 | 1 | 0 | 1 | 1 | 0 | 1 | 1 | 0 |
| 1 | 1 | 0 | 1 | 1 | 0 | 1 | 1 | 0 |
| 0 | 0 | 1 | 1 | 1 | 0 | 1 | 1 | 0 |
| 1 | 0 | 1 | 1 | 1 | 0 | 1 | 1 | 0 |
| 0 | 1 | 1 | 1 | 1 | 0 | 1 | 1 | 0 |
| 1 | 1 | 1 | 1 | 1 | 0 | 1 | 1 | 0 |
| 0 | 0 | 0 | 0 | 0 | 1 | 1 | 1 | 0 |
| 1 | 0 | 0 | 0 | 0 | 1 | 1 | 1 | 0 |
| 0 | 1 | 0 | 0 | 0 | 1 | 1 | 1 | 0 |
| 1 | 1 | 0 | 0 | 0 | 1 | 1 | 1 | 0 |
| 0 | 0 | 1 | 0 | 0 | 1 | 1 | 1 | 0 |
| 1 | 0 | 1 | 0 | 0 | 1 | 1 | 1 | 0 |
| 0 | 1 | 1 | 0 | 0 | 1 | 1 | 1 | 0 |
| 1 | 1 | 1 | 0 | 0 | 1 | 1 | 1 | 2 |
| 0 | 0 | 0 | 1 | 0 | 1 | 1 | 1 | 0 |
| 1 | 0 | 0 | 1 | 0 | 1 | 1 | 1 | 0 |
| 0 | 1 | 0 | 1 | 0 | 1 | 1 | 1 | 0 |
| 1 | 1 | 0 | 1 | 0 | 1 | 1 | 1 | 0 |
| 0 | 0 | 1 | 1 | 0 | 1 | 1 | 1 | 0 |
| 1 | 0 | 1 | 1 | 0 | 1 | 1 | 1 | 0 |
| 0 | 1 | 1 | 1 | 0 | 1 | 1 | 1 | 1 |
| 1 | 1 | 1 | 1 | 0 | 1 | 1 | 1 | 1 |
| 0 | 0 | 0 | 0 | 1 | 1 | 1 | 1 | 0 |
| 1 | 0 | 0 | 0 | 1 | 1 | 1 | 1 | 0 |
| 0 | 1 | 0 | 0 | 1 | 1 | 1 | 1 | 0 |
| 1 | 1 | 0 | 0 | 1 | 1 | 1 | 1 | 0 |
| 0 | 0 | 1 | 0 | 1 | 1 | 1 | 1 | 0 |
| 1 | 0 | 1 | 0 | 1 | 1 | 1 | 1 | 0 |
| 0 | 1 | 1 | 0 | 1 | 1 | 1 | 1 | 0 |
| 1 | 1 | 1 | 0 | 1 | 1 | 1 | 1 | 2 |
| 0 | 0 | 0 | 1 | 1 | 1 | 1 | 1 | 0 |
| 1 | 0 | 0 | 1 | 1 | 1 | 1 | 1 | 0 |
| 0 | 1 | 0 | 1 | 1 | 1 | 1 | 1 | 0 |
| 1 | 1 | 0 | 1 | 1 | 1 | 1 | 1 | 0 |
| 0 | 0 | 1 | 1 | 1 | 1 | 1 | 1 | 0 |
| 1 | 0 | 1 | 1 | 1 | 1 | 1 | 1 | 0 |
| 0 | 1 | 1 | 1 | 1 | 1 | 1 | 1 | 0 |
| 1 | 1 | 1 | 1 | 1 | 1 | 1 | 1 | 4 |

**Table S4**: Observed frequencies of allergic bronchopulmonary aspergillosis (ABPA) criteria patterns estimated by latent class analysis

| ABPA by 5 Patterson criteria | ABPA by 6 Patterson criteria | ABPA by 7 Patterson criteria | ABPA by 8 Patterson criteria | ABPA by Agarwal criteria | Observed frequency |
| --- | --- | --- | --- | --- | --- |
| Controlled asthma |  |  |  |  |  |
| 0 | 0 | 0 | 0 | 0 | 103 |
| 1 | 0 | 0 | 0 | 0 | 12 |
| 1 | 1 | 0 | 0 | 1 | 14 |
| 1 | 1 | 1 | 0 | 1 | 5 |
| 1 | 1 | 1 | 1 | 1 | 3 |
| Uncontrolled asthma |  |  |  |  |  |
| 0 | 0 | 0 | 0 | 0 | 172 |
| 1 | 0 | 0 | 0 | 0 | 29 |
| 1 | 1 | 0 | 0 | 0 | 1 |
| 1 | 1 | 0 | 0 | 1 | 19 |
| 1 | 1 | 1 | 0 | 1 | 10 |
| 1 | 1 | 1 | 1 | 1 | 4 |
